# Impact of the Crystal Structure of Silica Nanoparticles on Rhodamine 6G Adsorption: A Molecular Dynamics Study

**DOI:** 10.1021/acsomega.3c06657

**Published:** 2024-01-09

**Authors:** Daniel Doveiko, Karina Kubiak-Ossowska, Yu Chen

**Affiliations:** † Photophysics Group, Department of Physics, University of Strathclyde, Scottish Universities Physics Alliance, 107 Rottenrow, Glasgow G4 0NG, U.K.; ‡ Chemical Engineering, James Weir Building, University of Strathclyde, Glasgow G1 1XJ, U.K.

## Abstract

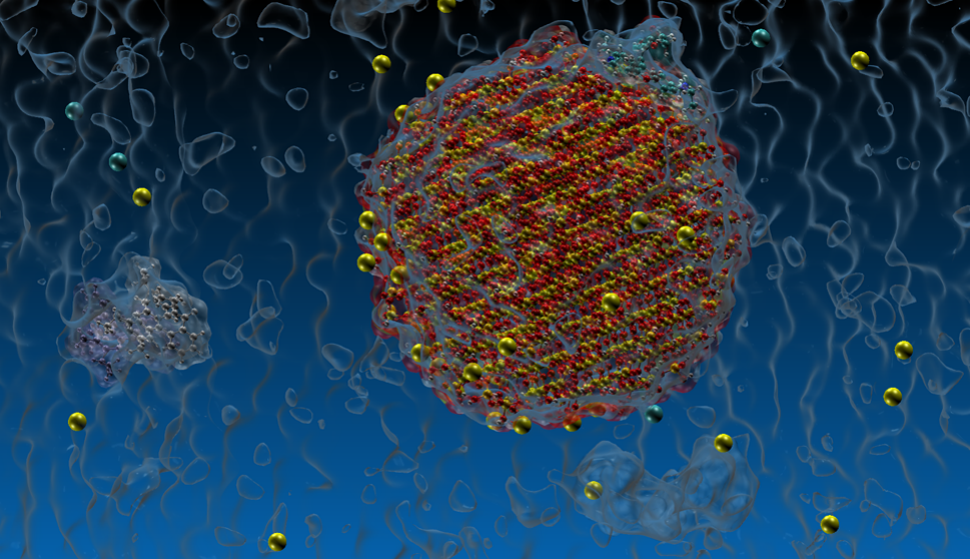

Understanding the mechanism of adsorption of Rhodamine 6G (R6G) to various crystal structures of silica nanoparticles (SNPs) is important to elucidate the impact of dye size when measuring the size of the dye–SNP complex via the time-resolved fluorescence anisotropy method. In this work, molecular dynamics (MD) simulations were used to get an insight into the R6G adsorption process, which cannot be observed using experimental methods. It was found that at low pH, α-Cristobalite structured SNPs have a strong affinity to R6G; however, at high pH, more surface silanol groups undergo ionization when compared with α-Quartz, preventing the adsorption. Therefore, α-Quartz structured SNPs are more suitable for R6G adsorption at high pH than the α-Cristobalite ones. Furthermore, it was found that stable adsorption can occur only when the R6G xanthene core is oriented flat with respect to the SNP surface, indicating that the dye size does not contribute significantly to the measured size of the dye–SNP complex. The requirement of correct dipole moment orientation indicates that only one R6G molecule can adsorb on any sized SNP, and the R6G layer formation on SNP is not possible. Moreover, the dimerization process of R6G and its competition with the adsorption has been explored. It has been shown that the highest stable R6G aggregate is a dimer, and in this form, R6G does not adsorb to SNPs. Finally, using steered molecular dynamics (SMD) with constant-velocity pulling, the binding energies of R6G dimers and R6G complexes with both α-Quartz and α-Cristobalite SNPs of 40 Å diameter were estimated. These confirm that R6G adsorption is most stable on 40 Å α-Quartz at pH 7, although dimerization is equally possible.

## 1 Introduction

Nanotechnology is an ever-growing field exploring the unique physical and chemical properties of constructs under 100 nm size. One of the fastest-growing nanotechnologies is the manufacture and use of nanoparticles that possess unique optical properties and a high surface-to-volume ratio^1,2^ and are widely used in nanomedicine and technology.^3^

Silicon is one of the most abundant elements on Earth, with around 78% of Earth’s crust consisting of various silicon and oxygen compounds, such as quartz, opal, and other silicates, in both crystalline and amorphous structures. Furthermore, silicon is present as silicic acid in the oceans and in some living organisms such as sponges and algae.^4^

Due to high abundance, silica nanoparticles (SNPs) are often used in scientific research and other industrial applications, such as drug delivery,^5^ various bonding and coating applications,^6^ agriculture,^7^ and many others.^8^ The properties of SNPs usually depend on their size; hence, it is crucial to have an accurate way of measuring it. Commonly used techniques include small-angle X-ray scattering (SAXS),^9^ small-angle neutron scattering (SANS),^10^ transmission electron microscopy (TEM),^11^ and dynamic light scattering (DLS);^12^ however, all of them have their drawbacks, namely, they are expensive^13^ and require complex sample preparations.^14^ Moreover, the aforementioned techniques might be inaccurate for particles under 10 nm size;^15^ therefore, a more precise method might be required for particular applications.

In the early 2000s, a new approach was proposed, utilizing the relationship between particle size and its rotational diffusion rate based on time-resolved fluorescence anisotropy of fluorescent dyes.^16^ The main disadvantage of this approach is the fact that SNPs do not exhibit strong intrinsic fluorescence; furthermore, the origin of this fluorescence is not entirely clear.^17^ Therefore, SNPs require additional labeling, and as a result, the measured size is not of the particle itself but rather the size of the SNP–dye complex. Moreover, because it is impossible to experimentally determine how the dye is oriented on the SNP surface, the dye contribution to the measured complex size is unknown.^18^ The above makes the precise determination of the nanoparticle size impossible.

One of the most promising dyes that can be used to label SNPs is Rhodamine 6G (R6G). R6G has a high quantum yield and possesses a remarkably high photostability^19^ and suitable fluorescence lifetime.^20^ Furthermore, its emission does not change when adsorbed to SNPs.^21,22^ Finally, the dye is cationic,^23^ resulting in electrostatic adsorption to SNPs without contaminating the samples with additional linking compounds. Nevertheless, due to the system size, employing an experimental approach to elucidate the details of R6G–SNP interactions is impossible.

Fortunately, the dye and SNP interaction mechanism can be explored using computational methods, such as molecular dynamics (MD), which allow full insight into processes on an atomistic scale. In this work, MD simulations were performed to elucidate the details of cationic R6G interactions with anionic α-Quartz and α-Cristobalite structured SNPs. The studies were designed in a way that allows the effects of SNP size and solute pH to be explored. The results presented here provide insight into the dye’s adsorption mechanism to the surface of the SNP, which can help determine the impact of the R6G size on the measured size of the SNP–dye complex. Additionally, they give insight into the role of the crystal structure of the SNP on the R6G adsorption, and the conclusions are very likely to be relevant to other anionic adsorbents. Moreover, the general interactions between R6G and SNPs can be potentially extrapolated to other fluorescent dye interactions with nanoparticles. As far as we know, this is the first MD study on fluorescent dye adsorption on SNPs.

In addition, we explored the possibility of R6G dimerization, which was previously studied by Dare-Doyen et al.^24^ and Chuichay et al.,^25^ taking into account two R6G molecules in the simulation system. We applied a more complex approach by examination of systems containing six R6G molecules in water only as well as in the presence of SNP(s) and solute ions, monitoring the aggregation process and measuring the binding energy of dimers using steered molecular dynamics (SMD). R6G is a cationic dye with a +1e charge at a wide pH range, and from the electrostatic point of view, the dye molecules should be unlikely to aggregate, although due to their geometry and resulting stacking interactions, aggregates might be potentially observed in water. Such aggregates can be potentially formed as a result of π–π interactions between two individual R6G molecules.^26^ Furthermore, it has been proven both experimentally^24,27,28^ and computationally that R6G creates stable dimers.^24,25^ As described below, even if multiple R6G molecules are present in the system, the highest stable aggregate is a dimer, and as in previous works,^24,25^ this does not require any ions mediating the R6G–R6G interactions, which further indicates the importance of van der Waals (VdW) interactions in atomistic simulations^29^ and provides additional cross-validation to our approach, models, and force fields (FF) used in our MD simulations.

## 2 Methods

**Figure 1:**
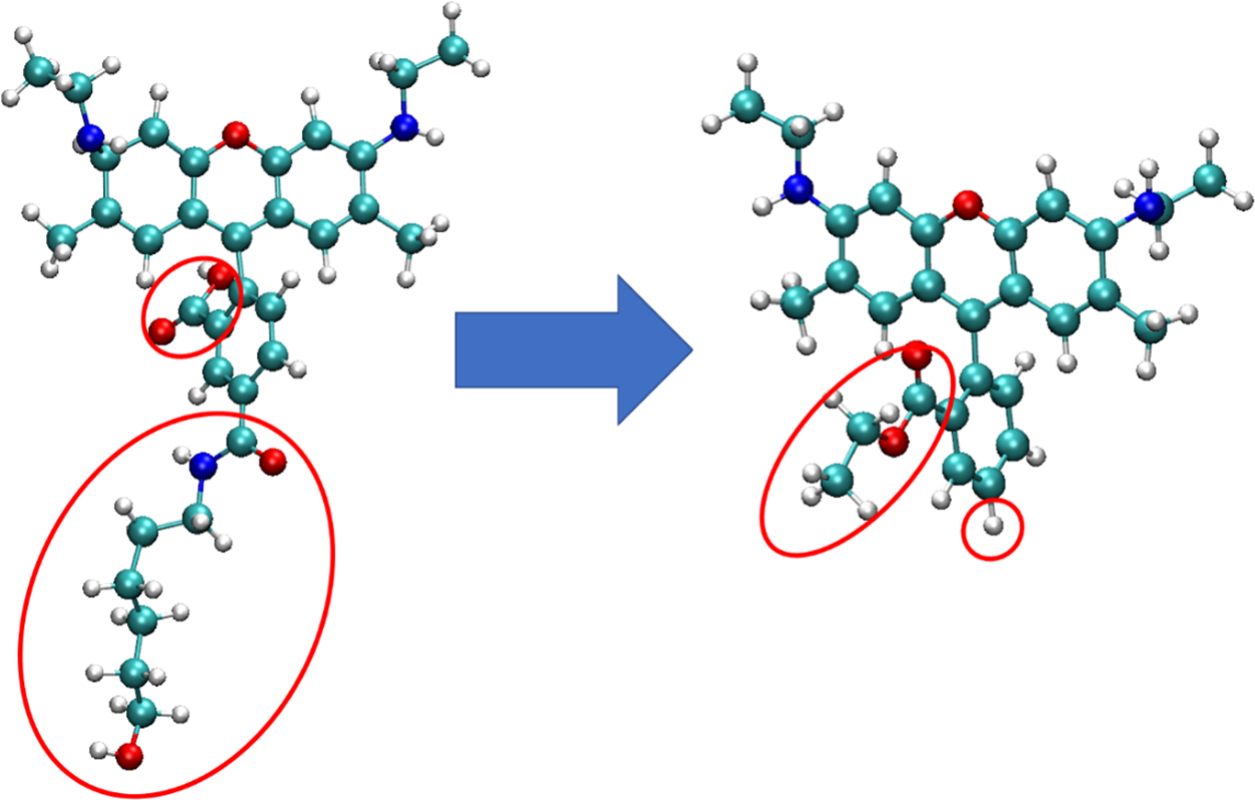
R6G structure from 2v3l.pdb (left) and the structure after modification, most commonly used in experiments (right) and therefore used in all MD simulations. Modified parts are circled in red.

The CHARMM–GUI interface^30^ was used to create the dye and SNP structures. The initial R6G structure was taken from the protein databank entry 2v3l.pdb.^31^ The dye structure was modified by removing the amino-alkyl tail and modifying the side chain as shown in Figure 1. The charges in CHARMM–GUI-generated topology files for R6G were manually corrected to match restrained electrostatic potential partial (RESP) atomic charges obtained from the highly accurate DFT B3LYP/6-31G** calculations reported by Chuichay et al.^25^ Finally, the generated force field (FF) parameters, including the chemical properties of R6G atoms, were compared with corresponding values obtained by Vaiana et al. using automated frequency matching^32^ for cross-validation. Furthermore, the R6G structure was previously used in other MD studies involving adsorption on gold electrodes^33^ and TiO_2_ hydroxylated surfaces.^34^

SNPs were built using the Nanomaterial Modeler extension in CHARMM–GUI.^30,35^ Two different crystal structures were used: α-Quartz and α-Cristobalite; for each 40 and 20 Å diameter SNPs were built at pH 7 and 12. While we refer to the SNPs as 40 and 20 Å diameter, due to their small size and effects of the crystal structure, the measured diameter between heavy atoms might slightly differ from the nominal value. The effects of pH are modeled through the degree of ionization (deprotonation) of surface silanol groups; we use 13.3 and 30% for pH 7 and 12, respectively. In all cases, the particles were built in vacuum.

**Figure 2:**
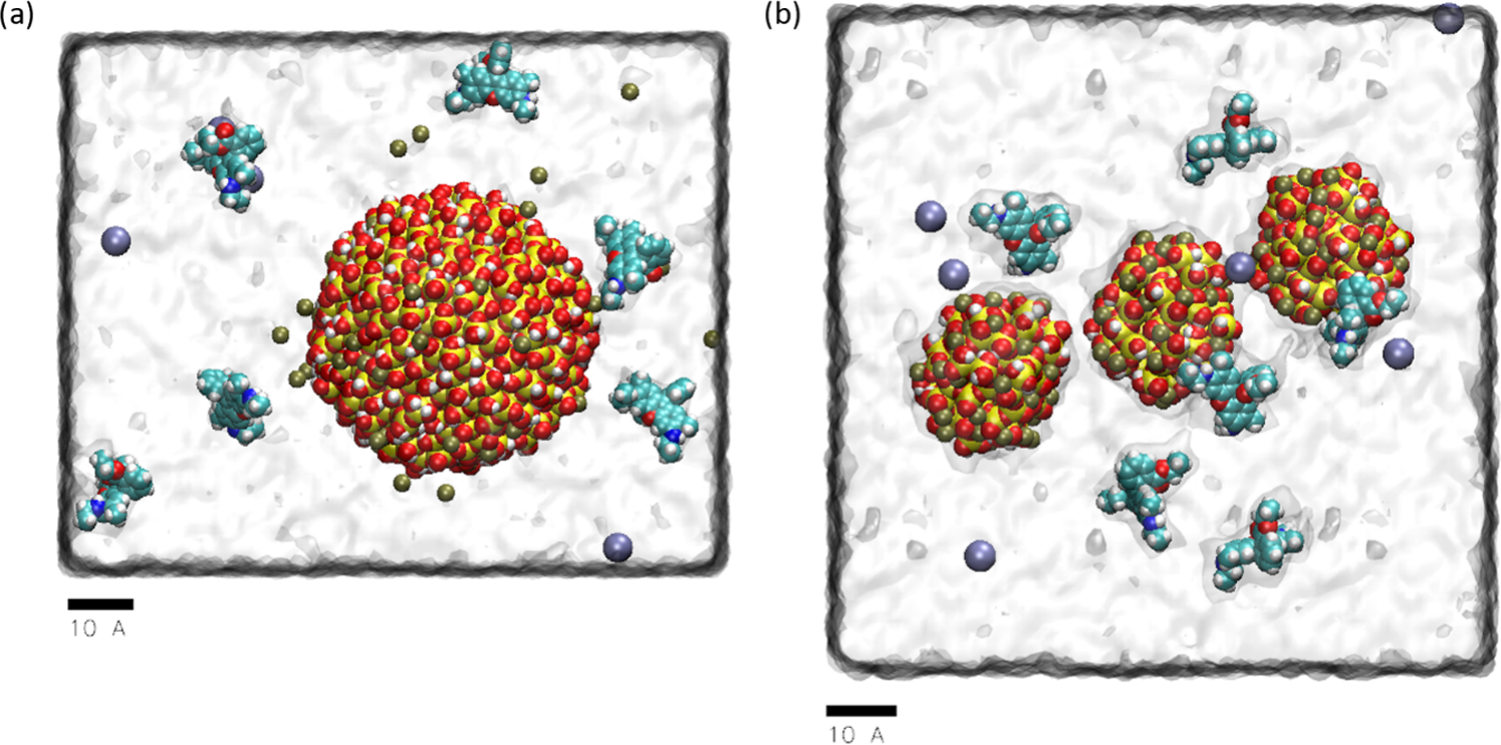
Initial system setup. (a) Example of 40 Å SNP system containing one SNP and six R6G molecules; (b) example of 20 Å system containing three SNPs and six R6G. Water is indicated by the transparent film, while oxygen (red), silica (yellow), hydrogen (white), carbon (cyan), chlorine (ice blue), and sodium ions (tan) are indicated by VdW spheres. Note the scale of each system.

The generated SNPs and modified R6G were uploaded into the “Multicomponent Assembler” of CHARMM–GUI. Each system contained six R6G molecules and one or three SNPs depending on the diameter (40 or 20 Å, respectively). The location and orientation of all system components were randomized. The systems were then solvated with TIP3P^36^ water and neutralized using VMD software.^37^ Six Cl^–^ ions were required to neutralize the cationic charge of R6G (+1e per molecule), while Na^+^ ions present in the systems came from the ionization of the SNPs according to the desired pH (see Table 1 for detailed system composition). The above resulted in a total of 8 systems, each containing six R6G molecules, one 40 Å α-Quartz or α-Cristobalite nanoparticle (both at pH 7 and 12) or three 20 Å α-Quartz/α-Cristobalite nanoparticles at both pH values. To distinguish the simulation systems, we introduced simplified names: 40qSNP7, 40qSNP12, 40cSNP7, 40cSNP12, 20qSNP7, 20qSNP12, 20cSNP7, and 20cSNP12 where the first number, 40 or 20, gives the diameter (in Å) of the SNP, symbol q or c stands for α-Quartz or α-Cristobalite, and the last number 7 or 12 indicates the pH. The initial system setup for 40 and 20 Å SNPs is visualized in Figure 2 and the total number of atoms per system was around 85,000.

**Table 1:** System Composition^a^

system	# of R6G	R6G	# of SNP	SNP	Cl	# of Na per NP/SNP charge	water (# molecules)	total
40qSNP7	6	64	1	3141	6	51	80,118 (26,706)	83,700
40qSNP12	6	64	1	3077	6	116	80,612 (26,871)	84,195
40cSNP7	6	64	1	2725	6	58	81,832 (27,277)	85,005
40cSNP12	6	64	1	2638	6	155	80,292 (26,764)	83,475
20qSNP7	6	64	3	436	6	11	82,731 (27,577)	84,462
20qSNP12	6	64	3	421	6	27	84,153 (28,051)	85,887
20cSNP7	6	64	3	376	6	17	83,325 (27,775)	84,894
20cSNP12	6	64	3	354	6	39	81,861 (27,287)	83,430

a From left to right: number of R6G molecules, number of atoms within one R6G molecule, number of SNPs added into the system, number of atoms involved in SNPs, number of Cl^–^ ions, number of Na^+^ ions, and number of water atoms.

All simulations were run using the NAMD3 CUDA version.^38,39^ Interface FF^40^ was used for the SNPs, while CHARMM36^41^ was used for the rest of the system. Interface FF is the extension of the most commonly used harmonic force fields such as CHARMM, AMBER, and GROMACS, and it allows the simulation of inorganic–organic and inorganic–biomolecular interfaces. This FF has been successfully used in MD studies involving organic compound interactions with various silica structures.^42−44^ As typical in MD simulations, the minimization of the system was done in two steps: (1) water only (1000 minimization steps and 100 ps equilibration in *T* = 300 K) and (2) the entire system (10,000 minimization steps followed by 30 ps of heating to 300 K and 270 ps of thermalization with 1 fs time step). In the production stage, the integration step was 1 fs, while the total length of the trajectory was 100 ns. Particle Mesh Ewald (PME) was used for the electrostatic interactions and VdW cutoff was set to 12 Å. For water, the TIP3P^36^ model was employed, while the internal water molecule vibrations were constrained. The anisotropic cell fluctuations ensured that the desired pressure of 1 atm at 300 K was reached and kept constant. Each production trajectory has been repeated four times from the same starting point to obtain better statistics and insight into the possible processes. It gave thirty-two 100 ns MD trajectories in total, all of which were carefully analyzed and the most representative trajectories or events are described herein. In all cases, there were no R6G interactions observed with the SNP image due to the primary simulation cell size.

The stable states of the representative MD trajectories were chosen for the starting configurations of the SMD simulations, namely, stable R6G–R6G dimer, R6G–40qSNP7, and R6G–40cSNP7 complexes. Most of the simulation parameters were kept as in standard MD, while the introduction of the external force with constant-velocity pulling required two additional parameters: pulling velocity of 0.01 Å/ps and harmonic constraint force constant of 4 kcal/ (mol Å) equivalent to 278 pN/Å. In all trajectories, one of the compound’s center of mass (COM) has been fixed to reduce the noise (coming from pulling the system in aqueous media, which causes constant creation and breakage of the hydrogen bonds between water and SNP particles) while the other compound has been pulled away. The force plot vs time and the compound displacement have been used to calculate the dissociation energies as described in Section 3.

Most of the analysis has been done using VMD software and combined with results obtained from a custom TCL script provided in the Supporting Information, which allowed for the extraction of COM (*x, y, z*) coordinates of the specified part of the system, as commonly used in MD analysis (e.g., see ref (45)). The COM distance plots for each SNP–R6G pair were created using MATLAB.^46^ The classification and differentiation between the adsorption event (state A), R6G reorientation close to SNP surface (state R/A), and simple electrostatic interactions, which do not result in adsorption but might trap the molecules in “adsorption-like” state (state T), have been made based on the COM distance and orientation of the molecules as visualized using VMD. Namely, a configuration has been identified as state A (adsorbed) if the distance between R6G COM and the SNP surface (marked as a gray line on COM plots) was not larger than 5 Å and simultaneously the R6G xanthene core was oriented parallel to the SNP surface. If R6G approached SNP, but its xanthene core never achieved a parallel orientation, the interaction was considered as a reflection of strong electrostatic interactions, which trapped the molecules in an “adsorption-like” state T. Typically, such events did not last longer than 2 ns, with the majority of them being under 1 ns. Finally, if the R6G xanthene core was oriented parallel, but the dye is repositioning on the SNP surface, this state is considered as reorientation state R/A. This state was usually combined with short adsorption periods.

## 3 Results and Discussion

In the case of each of the eight systems studied (40qSNP7, 40qSNP12, 40cSNP7, 40cSNP12, 20qSNP7, 20qSNP12, 20cSNP7, and 20cSNP12, using the notation from Section 2), the most representative trajectory of four repeats has been selected for the detailed description given below. Nevertheless, as Table 2 indicates, all of the trajectories show the same trend and are relatively similar; hence, any of the trajectories could be treated as the representative one. Due to the fact that the total number of silica particles differs between the systems, the trajectories are analyzed from an R6G viewpoint, as its concentration is the same in all systems. Having the above, it is possible to describe in detail the R6G adsorption mechanism to different SNPs under various conditions studied.

**Table 2:** Average Percentage of the Trajectory (for Each Repetition and Each System) for Which at Least One R6G Molecule is Adsorbed to the SNP Surface

repetition	40qSNP7 (%)	40qSNP12 (%)	40cSNP7 (%)	40cSNP12 (%)	20qSNP7 (%)	20qSNP12 (%)	20cSNP7 (%)	20cSNP12 (%)
1	31	3	30	6	53	3	38	10
2	20	21	24	0	29	21	32	7
3	21	12	28	9	51	12	37	5
4	18	0	48	0	24	0	23	0
average	23	9	33	4	39	9	30	8

### 3.1 Structural Differences between α-Quartz and α-Cristobalite Silica Nanoparticles

To be able to fully understand the impact of the crystal structure on the simulation outcome and R6G interactions with SNPs in particular, it is crucial to understand the structural differences between qSNPs and cSNPs and how those might affect the adsorption. Therefore, detailed composition of the SNP surface was analyzed to elucidate how the concentration of ionized silanol groups might affect the solute ion dynamics and in turn affect the electric field in the system.

**Figure 3:**
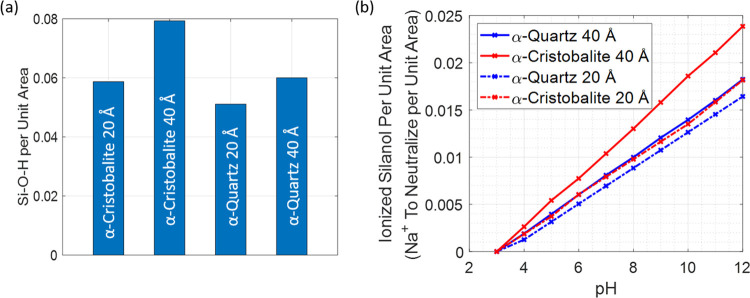
Silanol groups on the SNP surface. (a) Estimated number of surface silanol groups per unit area (Å^2^) and (b) number of ionized silanol groups per Å^2^ at different pH values. The figures were created by using the SNP structures at different degrees of ionization built by CHARMM–GUI. Afterward, by calculating the volume of the SNP of a specific size, the values per Å^2^ were estimated. Finally, the SNP sizes for both crystal structures were normalized to 40 and 20 Å to allow objective comparison.

Figure 3 indicates that the number of ionized groups per Å^2^ is always much higher in the case of α-Cristobalite, which has more surface silanol groups per Å^2^ when compared with α-Quartz of the same size. This comes from the fact that α-Cristobalite has a lower atomic packing fraction in the unit cell and molar density; hence, there are more silanol groups on the NP surface, which undergo ionization with growing pH.^47^ Details of the structures of qSNPs and cSNPs are visualized in Figure S1. A higher number of silanol groups with negative partial charges might indicate that there are more candidates to interact via electrostatic forces with the potential adsorbent; nevertheless, as is shown below, the entire picture is not so simple. Our simulations have been performed at pH 7 and 12, and the plot suggests that the strongest and most stable R6G adsorption should be on 40cSNP while the weakest on 20qSNP in both pH, and this effect should be more visible at pH 7. The adsorption stability should be comparable in the case of 40qSNP and 20cSNP at both pH values studied. However, this simple analysis does not entirely agree with the simulations that consider a more complex effect than only several ionized groups on the SNP surface. The simulation system has to be (i) neutral and (ii) exist in a buffer to reflect the experimental conditions; therefore, Na^+^ and Cl^–^ ions were added to the simulation cell. As Figure 3b indicates, the number of Na^+^ ions per Å^2^ required to neutralize the SNPs strongly depends on pH, while the dependence on SNP diameter is substantial in the case of α-Cristobalite but relatively minor in the case of the α-Quartz structure. The 40cSNP, which is characterized by the largest number of silanol groups requires the most Na^+^ to be introduced; the 20qSNP is on the opposite end, while 40qSNP and 20cSNP are in the middle and very similar. The presence of Na^+^ ions influences the accessibility of silanol groups to the adsorbent as well as the electrostatic field created by SNPs; therefore, even a slight discrepancy in the amount of Na^+^ per Å^2^ might cause a visible effect. The last might be analyzed by considering the dipole moment created by each SNP.

Dipole moments were measured using the built-in VMD “Dipole Moment Watcher” tool; however, due to precise measurement between well-defined moieties, it might not be possible to directly compare the obtained values with the experimental ones. Theoretical contemplation on usability of this tool for charged moieties is provided in the Supporting Information. At pH 7, the dipole moment for 20cSNP is 625 D (Debye), while for 40qSNP it is 925 D (∼50% difference). Similarly, at pH 12, it is 500 and 900 D, respectively (∼80% difference). For 20qSNP, the dipole moment is 125 D at pH 7 vs 250 D at pH 12 (100% difference), while for 20cSNPs, it is 100 vs 200 D (also 100% difference). The discrepancy in the dipole moment values explains the difference in adsorption observed at pH 7 where its affinity and stability are significantly higher in the case of qSNPs (as described in detail in the next section). However, at pH 12, the adsorption follows a different pattern as the dipole moments do not reflect the impact of the crystal structure of the SNP. As already mentioned, α-Cristobalite has a lower molar density, and as a result, cSNPs have more silanol groups on the surface. Therefore, at high pH, more of those groups will be ionized (deprotonated) with more Na^+^ introduced to the system. The counterions will comprise a labile layer on the SNP surface and reduce the R6G adsorption affinity (as visualized by VMD and shown in Figure S2) by exhibiting a repulsive force on the cationic dye.^48^ Lastly, it is a very nontrivial question whether to include or not include counterions into a cluster system during the calculations of dipole. We found that since sodium counterions are free to diffuse, and the dipole moment strongly depends on the distance; therefore; the values obtained in such a way would be strongly affected by ion diffusion. Hence, it would be difficult to extract the values of interest and more importantly it would make comparison between individual molecules impossible. In other words, the dipole moment has to be measured for SNP only, without the counterion layer, as the interacting ions moderate the electric field created by the SNP, resulting in the observed discrepancy.

An additional important factor is the effect of SNP size on the generated electric field. With decreasing SNP diameter, the size factor becomes less important as there are multiple SNPs in the system each with their own electric field. Therefore, R6G experiences a superimposed electric field created by multiple SNPs and R6G molecules present in the system. It is worth noticing that R6G molecules have a higher probability of adsorbing to cSNPs as the dye molecules favor binding to unionized silanol groups;^48,49^ nevertheless, the adsorption on qSNPs tends to be more stable and longer.

Recognition of the differences introduced by the internal structure and size of SNPs leads to a better understanding of the R6G adsorption mechanism under various pH values, which is described below in detail.

### 3.2 R6G Adsorption on 40 Å Quartz

**Figure 4:**
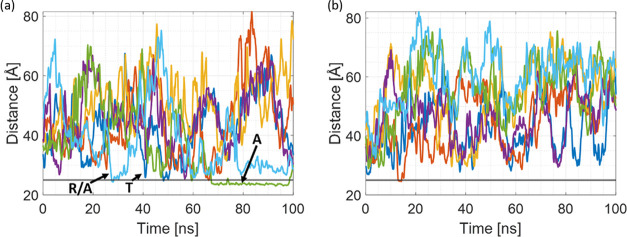
COM distance plots for (a) 40qSNP7 and (b) 40qSNP12. Fluctuating colored lines represent COM distances from each R6G molecule to the SNP COM, while the gray line represents the adsorption threshold, which is set as a 5 Å distance between the SNP surface and the R6G molecule.

Figure 4 shows the COM distance plot as a function of simulation time for each dye at pH 7 and 12. The exemplar trajectories are shown in Supporting Information Movies 40qSNP7.avi and 40qSNP12.avi. In the case of pH 7, the adsorption is significantly more stable and the overall time the dye is attached to the particle surface is significantly longer when compared with pH 12 (see Table 2), which is consistent with the previously discussed pH effect on 40qSNP as well as several Na^+^ per Å^2^ (Figure 3b), which is over two times smaller at pH 7 than at pH 12 (0.008 vs 0.018 of Na^+^ per Å^2^).

As already mentioned, the R6G adsorption stability is strongly impacted by the degree of ionization of the surface silanol groups and the formation of the counterion layer (see Figures S2 and S3). As discussed previously, at pH 7, the number of ionized groups is significantly lower than at pH 12; hence, the total number of Na^+^ ions, which screen the electric field created by the SNP is lower, and therefore, the adsorption is more probable and stable. However, at pH 12, where we have more ionized surface silanol groups, and as a result more Na^+^ in the system, there are more potential candidates to form the counterion layer that shields the electrostatic field. Additionally, the monomeric R6G adsorbs to the SNPs via the Si–O–H groups, forming hydrogen bonds.^48,49^ As a result, when more surface silanol groups become ionized and the SNP charge grows, the electrostatic attraction exerted on R6G also increases. Due to the high negative SNP charge, the Na^+^ ions form a layer on its surface, reducing the adsorption affinity. This, combined with the reduced amount of unionized silanol groups which are the primary location for binding, significantly suppresses the adsorption at high pH. For this reason, the analysis of R6G adsorption and its orientation on SNP presented below concentrates on results obtained at pH 7.

**Figure 5:**
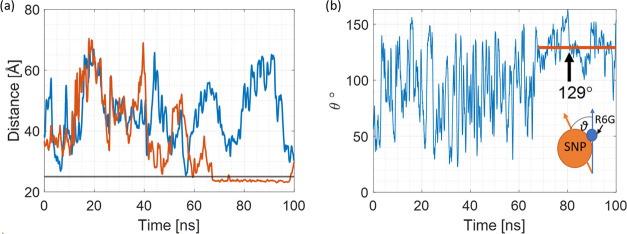
R6G adsorption process. (a) Simplified COM distance plot for two best adsorbing R6G molecules, R6G_4 (blue) and R6G_5 (red). The gray line marks the 5 Å distance from the SNP surface and (b) angle (θ) between SNP and R6G_5 dipole moments. The red line represents the average θ when R6G_5 is adsorbed. Inset in panel (b) shows how the θ angle was measured.

**Figure 6:**
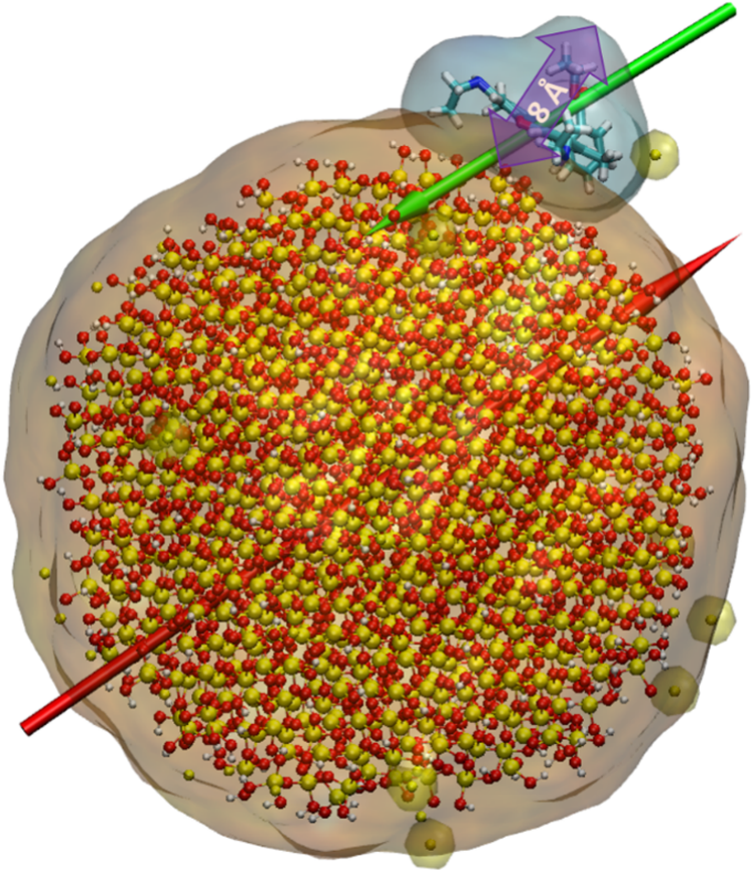
40qSNP7-R6G complex with visualized dipole moments. As predicted, in the case of state A, dipole moments are roughly in antiparallel orientation.

Figure 5a shows the simplified dye–NP COM distance plot as a function of the simulation time at pH 7 for the two best adsorbing R6G molecules to keep the plot clear. Initially, both R6G molecules (R6G_4 and R6G_5) are approximately 15 Å away from the SNP surface. After initial free diffusion, they form a dimer at around 15 ns that dissociates around 40 ns. At around 63 ns, R6G_5 adsorbs to the SNP surface and stays adsorbed until the end of the trajectory, which we describe as state A. In this specific case, the adsorption is very stable as the xanthene core is parallel to the SNP surface. As a result, due to this orientation, R6G has a ∼20% contribution to the measured R6G–SNP complex size as it lies almost perfectly flat with respect to the SNP surface (see Figure 6).

An alternative method of identifying the R6G state is monitoring the orientation of R6G and SNP dipole moments, which in the case of adsorption should be antiparallel (the angle θ between them should be 180°). It is important to note that an 180° angle might be achieved only in the ideal case of two isolated dipoles. In our case, the dipoles of interest are not isolated because each R6G molecule poses its dipole moment and the electric field is additionally modified by ions present in the system. The local electrostatics are extremely complex, and all subparts of the system are free to diffuse, including rotationally. Therefore, R6G of interest needs to constantly adjust its orientation to the fluctuations of the electric field around it. For this reason, θ values expected are in the range of 90 and 180°, and achievement of a stable angle of 180° is unlikely.

As illustrated in Figure 5b, the angle θ fluctuates between ∼25 and ∼150°, which is a result of SNP being almost stationary due to its large size and a free R6G that is moving freely. At around 63 ns, the situation calms down; θ changes more slowly and in a much smaller range. Between 63 and 100 ns, it fluctuates around a mean value of 129°, indicating that a stabilizing interaction was achieved. This observation combined with Figure 5a and visual analysis (40qSNP7.avi) indicates that R6G adsorbed onto the SNP, or in other words, state A (stable adsorption) was achieved. It is important to note that fluctuations of the θ angle value at state A are the reflection of the complicated electric interactions in the simulation system.

The adsorbed R6G molecule orients its xanthene core parallel to the SNP surface, while its tail protrudes from the surface and interacts with SNP via the flat part of the core. The thickness of the R6G xanthene core is ∼8 Å; therefore; the maximal contribution of R6G to measured SNP size is ∼20% (See Figure 6). It is worth noting that the multiple monomeric R6G adsorption was not observed due to the requirement of antiparallel dipole moment orientation of this nonsymmetric (at the xanthene core plane) molecule. This was observed in all our independent trajectories, i.e., eight systems with different sized SNPs at different pH values, over all four independent runs in each. Namely, adsorption on the opposite side of SNP would require either (i) adsorption of the xanthene core via the tail side with antiparallel dipole orientation or (ii) using the correct, flat side plane of the xanthene core with parallel dipole orientation. Nevertheless, as our results indicate, the R6G dipole moment needs to be antiparallel to the SNP dipole and R6G needs to expose its xanthene core to the SNP surface. In previous studies of R6G dimerization, the interactions were also via flat parts on the R6G molecule with the tails protruding from the dimer. Therefore, only one R6G monomer might adsorb to any sized SNP, or in other words, it is not possible to create the R6G layer on any sized SNP.

The above analysis indicates that R6G adsorption onto the SNP might be identified by (i) visual analysis of the trajectory (ii) monitoring the R6G–SNP COM distance and (iii) monitoring the angle between the dipole moment of each R6G molecule and the SNP. We performed the same level of analysis for all trajectories obtained and in the next subsections, the results regarding other crystal structures and SNP sizes are presented in the same way and order.

### 3.3 R6G Adsorption on 40 Å α-Cristobalite

**Figure 7:**
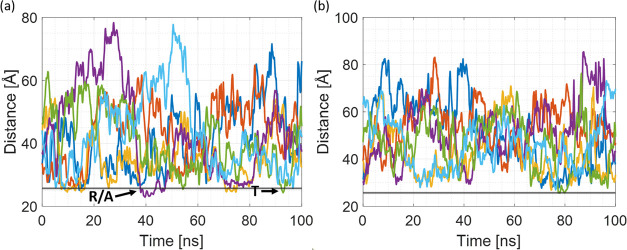
COM distance plots for (a) 40cSNP7 and (b) 40cSNP12. Fluctuating colored lines represent COM distances from each R6G molecule to SNP COM, the gray line marks the 5 Å distance from the SNP surface.

Figure 7 shows the COM distance as a function of simulation time for pH 7 and 12. For the case of pH 12 (Figure 7b), there is no absorption but only sporadically occurring stronger electrostatic interactions corresponding to state T. This can be identified from COM plots by looking at the time when R6G stays on the surface of the SNP. As we can see, in this case, the R6G molecules do not stay on the surface long enough to identify it as a state A, which corresponds to stable adsorption. Contrary to that, at pH 7, it is possible to identify some short adsorption periods (state R/A as described below). This observation is again in line with the number of Na^+^ per Å^2^ (Figure 3b), which is ∼2.5 times smaller at pH 7 than at pH 12 (0.0104 vs 0.0239), and the exemplar trajectories are visualized in Supporting Information Movies 40cSNP7.avi and 40cSNP12.avi.

As in the case of 40qSNPs, for the in-depth analysis, the COM distance plot for the pH 7 case was selected and simplified by keeping only those R6G molecules that show the strongest interactions with 40cSNPs.

**Figure 8:**
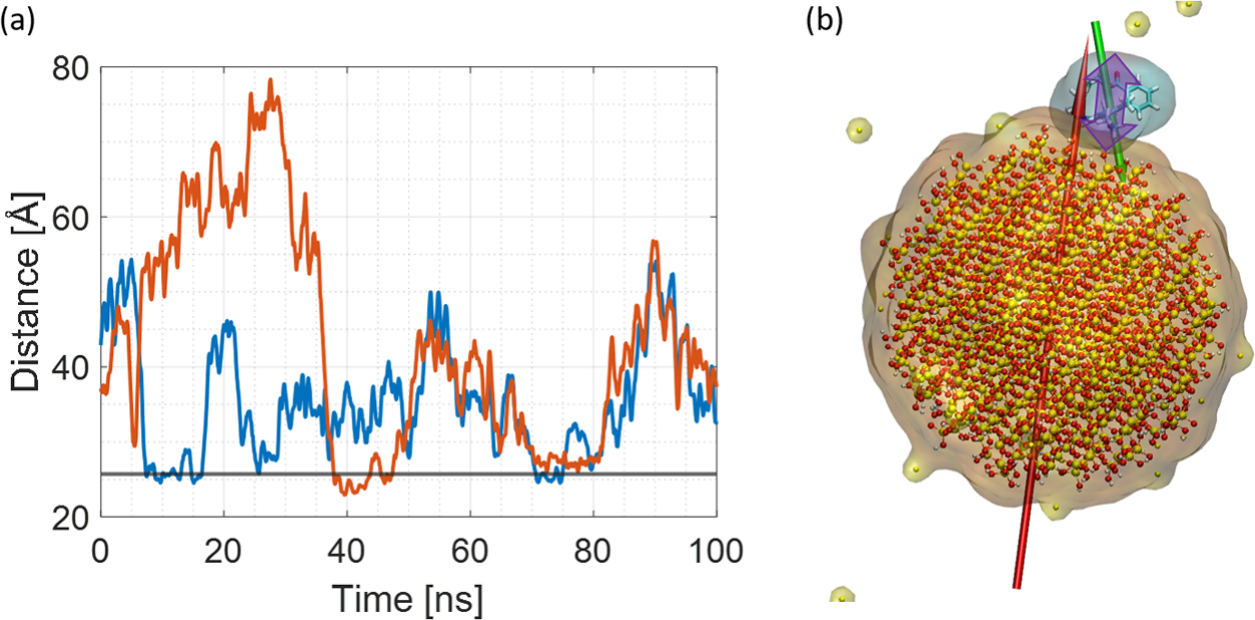
R6G adsorption on 40cSNP7. (a) Simplified COM distance plot for two best adsorbing R6G molecules, R6G_3 (blue) and R6G_4 (red); the gray line marks the 5 Å distance from the SNP surface. (b) 40cSNP7-R6G complex with visualized dipole moments.

Figure 8a shows the COM distance as a function of simulation time for R6G_3 and R6G_4 at pH 7. Similar to 40qSNP7, in the case of 40cSNP7 systems, the dye molecules were initially more than 12 Å away from the SNP surface, which indicates there are no nonbonded interactions, which would bias the system and/or drive R6G molecules toward adsorption. After a short, ∼3 ns period of free diffusion in the electrostatic field sourced by 40cSNP and modified by solute ions, R6G_3 adsorbs onto the SNP surface where it stays for 16 ns (until the 19th ns). The next adsorption event happens at around 39th ns when after long free diffusion, R6G_4 adsorbs to the surface of the SNP and stays adsorbed until the 43rd ns when it experiences interactions with R6G_3, forms a dimer at 50 ns, and desorbs (see Supporting Information Movie 40cSNP7.avi). It is important to note that both adsorption events are not very stable and therefore are identified as states R/A but not state A as in the case of 40qSNPs. This is because, on the COM distance plot, the reported adsorption events are represented as multiple and relatively short interactions. A closer look at the plots indicates that during the events observed at the periods 3–19 and 39–43 ns, there are notable distance fluctuations that contradict our definition of state A (stable adsorption). Nevertheless, the plots and visualization of R6G behavior suggest that this is state R/A, when, according to our definition, the R6G molecule is near the SNP surface (so it might seem to be weakly adsorbed), but the orientation of the xanthene core is being changed. Although the adsorption is not as stable as in the case of 40qSNP7, the R6G_4 xanthene core is still oriented parallel between 39 and 43 ns. Because the sizes of 40qSNP and 40cSNP are almost identical, the R6G also has a ∼20% contribution to the measured R6G–SNP complex size, as in the case of 40qSNP7.

The events observed from 50th ns until the end of the trajectory are related to R6G dimer interactions rather than a monomer. During this period, R6G_3 and R6G_4 are dimerized (details regarding R6G–R6G interactions are given in Section 3.7); hence, the interaction with SNP observed between 70 and 80 ns cannot be classified as monomer adsorption. During this time, the R6G dimer approaches the SNP at a distance suggesting possible adsorption of monomeric R6G. Nevertheless, due to the competition between forces governing the adsorption and those holding the dimer together, any of the R6G molecules can orient its xanthene core parallelly to the SNP surface. However, as the flat parts of the core face the other R6G molecule and not the SNP, we do not observe dimer adsorption on SNPs due to the geometric restraints, where the R6G xanthene core has to be oriented parallel to the SNP surface. Hence, although the dimeric R6G might spend some time close to SNP, the R6G dimer adsorption is not possible because the optimal electrostatic and geometry are not possible to achieve. Moreover, the R6G–R6G VdW forces are weaker than SNP–R6G electrostatic attraction, and as a result, we can see dimer dissociation and adsorption of monomeric R6G to the SNP in addition to dimer–SNP interaction and desorption.

The angle between the SNP dipole moment and R6G_3/R6G_4 dipole moment is plotted in S4. In this case, the θ angle fluctuations are substantial during most of the trajectories; therefore, achievement of state A is excluded. Nevertheless, it is possible to find two short periods when the angle seems to be a little more stable at large values (close to 150°): R6G_3 between 11 and 18 ns fluctuates around 131°, while R6G_4 between 39 and 44 ns fluctuates around 135°. Those periods overlap with R/A states as identified based on COM distance plots (Figure 8) and suggest that the θ angle between R6G–SNP dipoles is achieving orientation, which is close to the antiparallel one. As stated before, the short period indicates that those states should be classified as R/A ones (see movies 40cSNP7.avi and 40cSNP12.avi).

### 3.4 R6G Adsorption on 20 Å α-Quartz

In the experiments involving SNPs, including those with R6G-labeled SNPs, the system might be polydispersed and contain SNPs of various sizes and crystal structures.^50,51^ Therefore, the effect of the size has been examined and summarized below. The main difference between 40 and 20 Å SNP systems is the number of silica nanoparticles in the simulation, one and three, respectively.

**Figure 9:**
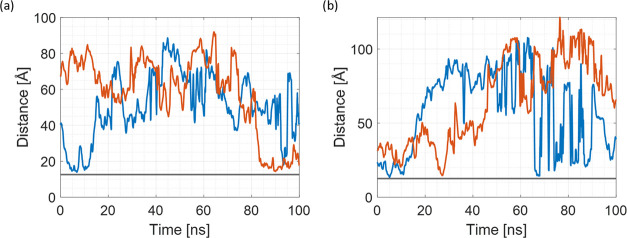
COM distance plots for 20qSNPs. (a) Simplified COM distance plot for two best adsorbing R6G molecules, R6G_1 (blue) and R6G_2 (red) for 20qSNP7 and (b) simplified COM distance plot for two best adsorbing R6G molecules, R6G_1 (blue) and R6G_2 (red) for 20qSNP12. The gray line marks the 5 Å distance from the SNP surface.

Figure 9 shows the most representative of R6G–SNP interactions COM distance plots obtained for 20qSNP7 and 20qSNP12. The exemplar trajectories are provided in the Supporting Information (20qSNP7.avi and 20SNP12.avi). The major difference between 20SNPs and 40SNPs is the lack of state A at pH 7, which might be explained by a smaller amount of silanol groups per Å^2^ (0.051 vs 0.06), as shown in Figure 3a.

In the case of 20qSNP7, adsorption is represented as a series of multiple short interactions with R6G repositioning spontaneously, which corresponds to state R/A. As shown in Figure 9a, the R6G–20 Å SNPs COM distance never goes beyond the “stable adsorption” distance of 5 Å to the SNP surface. Furthermore, R6G molecules stay far away from any SNP for a longer time than was observed in the case of 40 Å SNPs. It might be a result of the more complicated electrostatic field with more sources. The electrostatic field exerted on particular R6G molecules is now a superposition of electric fields created by all three nanoparticles, other R6G molecules, and solute ions. To adsorb/interact with any SNP R6G needs to be well-oriented with respect to all three SNPs while prioritizing one of them; hence, the lag time is longer so the entire process slows down significantly. Furthermore, 20 Å SNPs diffuse faster than 40 Å ones; therefore, the field fluctuations are larger, which additionally impacts the adsorption speed. Lastly, the charge of 20 Å SNP is significantly lower than 40 Å ones, resulting in a lower electrostatic attraction to that specific SNP (see Table 1 for system details) and the dipole moment values discussed in Section 3.1.

At pH 12 (system 20qSNP12), neither the adsorption state A nor R/A is detected; the COM distances are not stabilizing in any case (Figure 9b). The same has been detected based on θ angle analysis (data not shown). Events visible for R6G_1 during the first 10 ns of the trajectory and for R6G_2 around 27 ns correspond to state T when according to our definition R6G approaches the SNP for a short time, but it is not able to achieve the orientation allowing for establishing strong interactions; hence, the electric field fluctuations drive it away from the SNP in a short period. The same applies in the case of events detected for R6G_1 after 65 ns of the trajectory. A similar situation has been observed and confirmed by visual analysis in all four repetitions of the trajectories; therefore, the θ angle plots are not presented.

When comparing 20qSNPs7 with 40qSNPs7, it can be concluded that the particle size has a significant impact on the stability and rate of adsorption as mentioned in Section 3.1. Trajectories obtained for systems containing 40 Å SNP, which possess higher negative charge than 20 Å ones (−51e vs −11e), exhibit more stable adsorption; therefore, the majority of the events correspond to state A. Fast diffusion of 20 Å SNPs is another factor depreciating its role as a stable adsorption seed.

### 3.5 Adsorption to 20 Å α-Cristobalite

Finally, we will look at the adsorption to 20 Å α-Cristobalite following the same methods as in previous sections. Figure S5 shows the COM distance evolution as a function of simulation time for 20cSNP7 and 20cSNP12 (exemplar trajectories are shown in Supporting Information Movies 20cSNP7.avi and 20cSNP12.avi). Similar to 20qSNPs at pH 7, the R6G–SNP interactions are significantly shorter and less stable in the case of small particles; COM plots (Figure S5) report only one state R/A detected for R6G_6 during the last 10 ns of the trajectory. It is worth mentioning that the picture obtained for 20cSNP12 is analogous to 20qSNP12, with only state T detected.

Furthermore, the 20cSNP12 case illustrates well the domination of VdW forces responsible for R6G dimerization over the electrostatics forces responsible for its adsorption. In all trajectories obtained for this setting, much more dimerization events than adsorption-related ones occurred. More specifically, molecules R6G_2 and R6G_5 form a dimer at around the 36th ns until the 80th ns, while there are no states A or R/A present during the trajectory (see Figure S5 for details). As already mentioned, in the case of α-Cristobalite SNPs, more surface silanol groups are ionized when compared with α-Quartz particles of the same size (0.018 vs 0.016, see Figure 3) complicating the adsorption. As a result, the VdW interactions are dominant at high pH and the R6G molecules favor dimerization over adsorption.

As in the case of 20qSNPs, due to the lack of states A and R/A, it is impossible to identify the adsorption events by measuring the angle θ, and as a result, this method is not explored in this system as well.

### 3.6 System Comparison

When comparing 20SNPs with 40SNPs, there are a few notable differences in the adsorption of R6G. First, since smaller particles can diffuse faster, the probability of adsorption is lower; therefore, the majority of the interactions are classified as states R/A. Second, each particle in the case of 20SNPs exhibits its Coulombic force on the R6G molecule, interfering with potential adsorption to other SNPs. Finally, the curvature of the particle has to be taken into account. In the case of larger SNPs, R6G molecules will lay flatter when the xanthene core is parallel to the surface as it is in the case of 40qSNP7. However, if the SNP is more curved (20SNPs), then the dye adsorbs only via its xanthene core and the end tail floats freely. As a result, with more curved and less spherical particles, the contribution of the dye to the measured complex size is notably larger when compared with more spherical and less curved particles. Lastly, we need to mention the potential possibility for SNP interactions in the systems containing multiples of those. Although the system composition is very different when comparing 20 Å systems containing three SNPs and 40 Å systems containing only a single SNP, in the current setup and the used SNP and dye concentrations, we did not observe any significant nanoparticle interactions, which would strongly impact the R6G interactions with the SNPs of interest. The main factors that had a dominating effect on the mechanism of adsorption were the crystal structure, pH, and size of the SNPs and not the number of those in the system.

It is important to note that for smaller particles, the R6G size has a significantly higher impact on the measured size of the SNP–R6G complex. Due to the small size of the SNPs, the direction in which the diameter of the SNP is measured and the location where the dye adsorbs will have a significant effect. Although the xanthene core is oriented parallel facing the SNP surface, due to the larger curvature of the 20 Å SNP, it looks as if R6G protrudes more significantly (see Figures S7, 6, and 8). Additionally, 20SNPs are not perfectly spherical; as a result, the measured diameter will vary slightly depending on the measurement direction and the place where the dye molecule is adsorbed, the R6G size contribution to the measured complex size can be up to 30% in such constructs, while in the case of 40SNPs it is ∼20%. The size comparison for pH 12 is omitted as we do not observe any A or R/A states in those cases. It is important to note that the R6G size that is added to the SNP is no larger than 8 Å, which is particularly important for SNPs under 10 nm size, that are the main subject of this work and whose size cannot be measured accurately using conventional techniques. Nevertheless, as already mentioned, independent of the size, structure, and pH, only one monomeric R6G might adsorb onto the SNP and the R6G layer formation is not possible due to the requirement of (i) antiparallel orientation of the dipole moments and (ii) exposure of the xanthene core of Rhodamine 6G toward the SNP surface.

Furthermore, at pH 7, cationic R6G adsorbs better to cSNPs due to a higher number of surface silanol groups when compared with qSNPs (0.0079 vs 0.0069 for 20SNPs and 0.010 vs 0.008 for 40SNPs). However, at high pH, the number of ionized groups grows significantly faster with growing pH when compared with qSNPs, resulting in a decrease in the adsorption affinity. This can be seen well by looking at average values when the dye is adsorbed to the particles for 40SNPs in Table 2, where at pH 7, the average time R6G is adsorbed to the SNP is higher (33% for cSNP vs 23% in qSNP). At pH 12 however, as the cSNP undergoes more significant ionization, the adsorption affinity drop is much more significant (8.25 times in cSNPs vs 2.5 times in qSNPs). Lastly, we need to mention the structural difference of the SNPs. As discussed in Section 3.1, α-Cristobalite has a lower molar density and lower atomic packing fraction when compared with α-Quartz, due to which the cSNPs are less spherical and the measured diameter is strongly impacted by the measurement direction when compared with qSNPs, although they have fewer atoms in their structure, e.g., 20qSNP7 have 436 atoms per SNP while 20cSNP7 have 376 (see Table 1 for explicit system details).

### 3.7 Rhodamine 6G Dimer Formation

**Figure 10:**
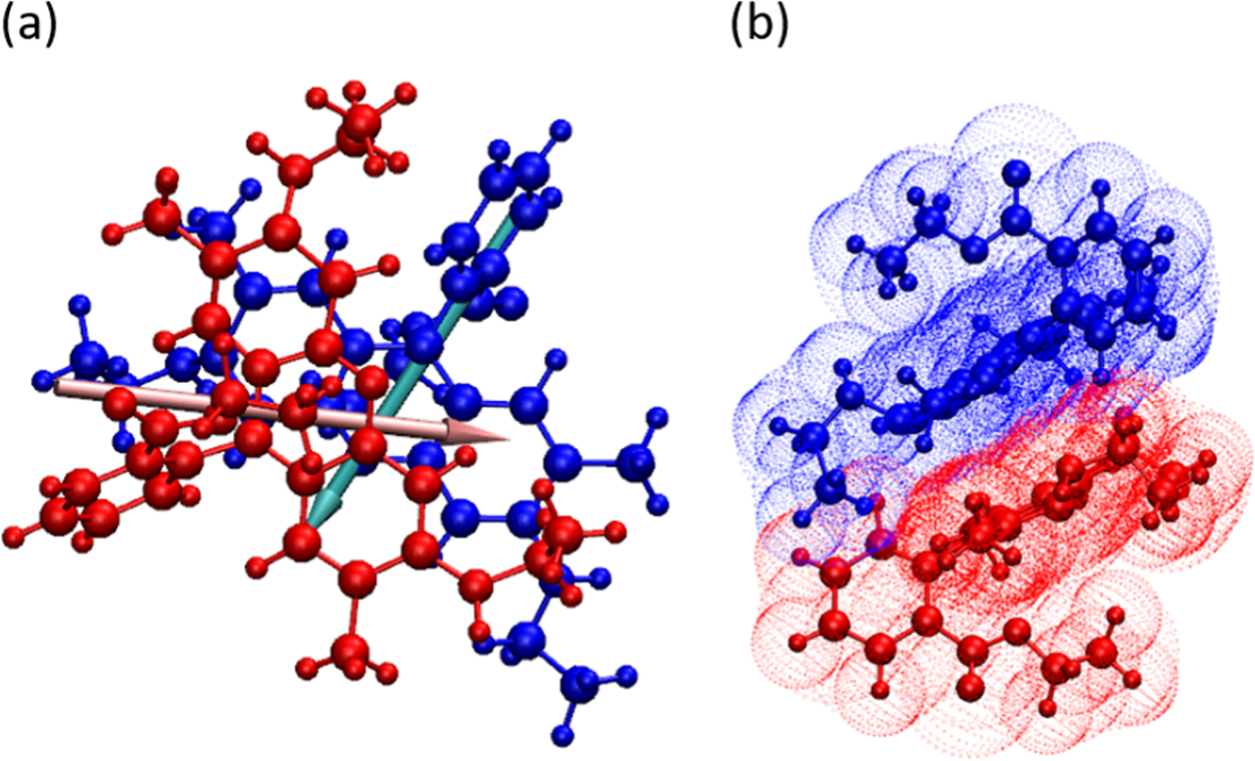
R6G Dimer: (a) top view of the dimer with visualized dipole moments and (b) side view of the dimer.

It is well-known that if used in high concentration or in the presence of silica films, R6G tends to form dimers.^52−54^ The dimerization mechanism is not well understood as both R6G molecules possess +1e charges that repel each other. However, the dimer is formed without the incorporation of any counterions mediating R6G–R6G interactions (Figure 10). As it was already reported elsewhere,^25,26^ the potential explanation for dimer formation is π–π stacking, which refers to orbital overlap between the pi bonds of aromatic rings, that has a strong binding force and often imposes geometric constraints.^55^ As a result, the geometry of observed R6G dimers may be dictated by the overlaps of those orbitals. Furthermore, our obtained dimer configurations match those that were reported previously.^24,25^ Nonetheless, the π–π interactions cannot be quantitatively estimated using classical MD simulations as they are not explicitly calculated during simulation; however, they are incorporated in the VdW parameters and therefore it is possible to observe the dimer formation in MD simulations.

We have noticed that R6G adsorption on SNPs and R6G dimerization are competing processes. R6G monomers tend to adsorb on SNPs while dimers do not. Interestingly (as visualized in Supporting Information DimerDesorption_40qSNP7.avi), the adsorbed R6G monomer might be approached by other R6G monomers that lead to dimerization and desorption. Alternatively, we can see a competition between the dimer and monomer, where a monomer can approach a dimer, temporarily creating a trimer and replacing one of the dimer components. Usually, such trimers did not exist for more than 8 ns (as visualized in R6GTrimer.avi). Another possibility is the dimer interaction with the SNP, which is followed by dimer breakage and resulting in one adsorbed R6G and one free R6G molecule (as visualized in Supporting Information DimerDissociation_40qSNP7.avi). Both desorption and dissociation were observed for 40qSNP7, 20qSNP7, and 20qSNP12 systems, while desorption only in the 40cSNP7 system and a single event of dimer dissociation (breakage) only in the 20cSNP7 system. In other words, our trajectories confirm that R6G might form stable dimers both in solute and on the SNP matrix, while trimers are rather intermediate states and reflect the possibility of molecules exchange. Summarizing R6G might exist as (1) free monomer, (2) monomer adsorbed on the SNP, and (3) free dimer, while conglomerates such as the adsorbed dimer, free and adsorbed trimer are not stable, intermediate states. Both the trimer and adsorbed dimer are unstable due to geometric constraints, i.e., for the stable adsorption/dimerization to occur, the xanthene core of R6G must be oriented parallel to the SNP/other R6G molecule, which is a dimer component. The obtained trajectories confirmed the existence of a mixture of the above moieties at the time.

It is worth emphasizing that there is no apparent reason why the last would not apply to larger than 40 Å SNP and silica surfaces. Furthermore, in the case of high R6G concentration, dimerization in solute is favored over dimerization on the SNP matrix independent of the crystal structure of the SNP in the system.

To get a better insight into the process of dimerization, we monitored this process in all of the systems in each repetition and calculated the number of dimers in the solute and on the SNP matrix as listed in Table 3. Due to the temporal character of trimers, they are not included in the analysis.

**Table 3:** Dimer Statistics for All Systems Averaged over Four Independent Runs of Each Trajectory^a^

	40qSNP7	40qSNP12	40cSNP7	40cSNP12	20qSNP7	20qSNP12	20cSNP7	20cSNP12
⟨Dimer⟩	4.75	3.25	5	3.25	4.75	5.75	3.75	3.5
⟨*T*⟩ (ns)	82.22	81.65	106.13	90.18	80.95	115.6	77.22	94.53
⟨*T*_PerDimer_⟩ (ns)	18.69	26.38	23.68	27.42	20.97	21.17	29.31	25.04

a ⟨Dimer⟩ indicates the average number of dimers in the system, ⟨*T*⟩ indicates the sum of the time the dimers existed in the system, and ⟨*T*_PerDimer_⟩ indicates the average time the given dimer existed in the system before dissociating.

For 40SNP systems, there were 60 dimers formed in the solute and five dimers formed on the SNP matrix, all in pH 7. Out of those five dimers, three were formed in the 40qSNP7 system and one of them dissociated, while two desorbed, and another two dimers were formed in the 40cSNP7 system and both desorbed before dissociating. In 20SNP systems, 64 dimers were formed in the solute and 7 were formed on the SNP matrix. The latter was present in all systems, except 20cSNP7, two in 20qSNP7 with one of them dissociating, four in 20qSNP12 two of which dissociated, and a single dimer in the 20cSNP7 system that also dissociated. Furthermore, we have noticed that for 40SNPs, independently of the crystal structure, the average amount of dimers in the system drops at pH 12 when compared with pH 7, e.g., 4.75 in 40qSNP7 drops to 3.25 in the 40qSNP12 system (see Table 3). It is important to note that although the number of dimers goes down with growing pH, the dimer stability increases, with the average time a dimer exists in the 40qSNP system growing from 18.69 ns in pH 7 to 26.38 ns in pH 12. The same trend is observed for the 40cSNP systems for both the number of dimers and the time of existence. When looking at the 20qSNP case, we can see that the trend is somewhat different. In the case of smaller SNPs, the average number of dimers increases from 4.75 at pH 7 to 5.75 at pH 12, and the time of existence also grows, from 20.97 to 21.17 ns. This suggests that in addition to the pH, the SNP size also has an impact on the process of dimerization. It might be speculated that with the rising diffusion speed of SNPs, it is more difficult for R6G molecules to achieve the orientation favoring the adsorption; hence, they are more prone to other possible processes, namely, the dimerization. The counterion layer density at high pH is another factor that leads to enhanced dimerization.

Close examination of the dimer behavior in all of the systems indicated that for 40qSNP7 systems, the dimer dissociation tends to happen faster than the whole dimer desorption (7.76 vs 12.7 ns), while for 20qSNP7 and 20cSNP12 systems, the dissociation is significantly slower when compared with desorption (3.4 vs 18.8 and 9.6 vs 13 ns). Looking at the results mentioned above, we can conclude that the time of desorption/dissociation is strongly affected by the size and/or the number of SNPs in the system. We have found that with bigger SNPs, the adsorption is more stable and we are more likely to reach state A; hence, one of the dyes forming a dimer is more likely to adsorb to the surface. As the binding energy for R6G–qSNP is higher than that of R6G–R6G, the dimer dissociates, leaving behind an adsorbed R6G. On the other hand, when the system contains multiple small SNPs, the R6G molecule cannot reach state A even in its monomeric form. As a result, it takes significantly longer for one of the components of the dimer to reach an optimal orientation, which would result in dimer dissociation and adsorption.

**Figure 11:**
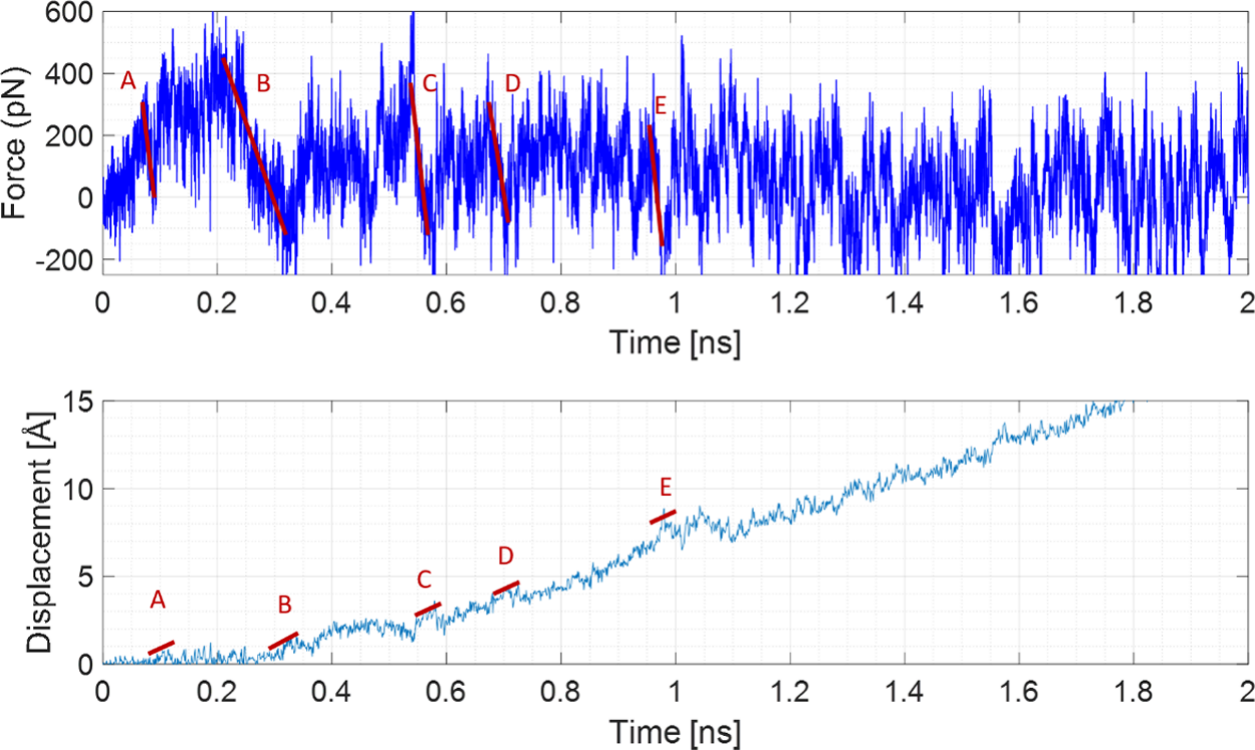
Force and displacement as a function of time for the R6G pulled from 40qSNP7 with constant velocity. Desorption steps (A–E, red lines) are labeled.

To compare the dimer binding energy vs adsorption energy of the monomer, constant-velocity pulling SMD simulations were performed, which allowed us to monitor the forces that can be monitored via AFM experiments. Unfortunately, such experiments cannot be performed for our system due to its small size. Therefore, we performed SMD with constant-velocity pulling to understand which interaction is stronger, adsorption or aggregation. Here, we show the most representative SMD simulations obtained by pulling R6G away from the 40qSNP7 and 40cSNP7 surfaces (the SNPs were fixed in position) at pH 7 (movies R6GqSNP_SMD.avi and R6GcSNP_SMD.avi). Additionally, the RG6 dimer created at pH 7 has been pulled apart, and again one molecule has been fixed (movie DimerSMD.avi). Fixing one of the molecules involved in the interaction led to noise reduction in the force plots, although the noise level has still been considerable (Figures 11, S6, and S7).

Both R6G desorption from the surface of SNPs and dimer dissociation was a multistep, gradual process. By looking at the force and displacement graphs as a function of simulation time (Figure 11), and using VMD for cross-checking if the force drop and displacement increase are related to an event that looks like a part of the desorption/dissociation process, the binding energies d*E* have been estimated using the potential energy of the spring formula
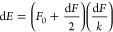
1where *F*_0_ is the force at the end of the transition, d*F* is the force change during the transition, and *k* is the spring constant. This method has already been successfully used for estimating the desorption energies of proteins^56−58^ and has shown reasonable agreement with experimental results. More detailed consideration is provided in the Supporting Information. Having *k* = 278 pN/Å and calculating d*E* for each of the transitions, we calculated Δ*E* related to dimer dissociation/desorption energy as a sum of all d*E*s. The energy required for R6G desorption from 40 Å α-Quartz SNP is ∼1.08 eV (Figure 11), desorption from 40 Å α-Cristobalite requires ∼0.36 eV (Figure S8), while the dimer dissociation requires ∼0.27 eV (Figure S9). Although it was not possible to estimate reliable energy barriers for R6G rotation, the obtained energy values further confirm our findings from the individual 40SNP studies, where qSNPs have the highest adsorption affinity, cSNPs have lower, and the weakest interactions in the system are between two R6G molecules forming a dimer. Nevertheless, we need to point out the SMD results are preliminary, and the systematic SMD experiments potentially combined with umbrella sampling are planned to confirm the above.

## 4 Conclusions

In this work, MD simulations were used to study the effect of the SNP crystal structure at pH 7 and 12 on the adsorption of R6G. In total, 32 independent 100 ns trajectories were simulated and analyzed, and the time the dye is adsorbed to the SNP was estimated. It was found that due to lower molar density, α-Cristobalite has more silanol groups on the surface that aid adsorption when compared with α-Quartz (0.08 vs 0.06 for 40SNPs and 0.06 vs 0.055 for 20SNPs); however it was found that qSNPs tend to have stronger and more long-lasting adsorption. Furthermore, at higher pH, more surface silanol groups are ionized in cSNPs when compared with qSNPs (0.024 vs 0.018 for 40SNPs and 0.018 vs 0.016 in 20SNPs) due to which the adsorption is almost nonexistent at high pH, suggesting that high pH α-Quartz is a better structure. Lastly, it was found that the stable adsorption (state A) occurs only when the engaged molecules’ dipole moments are antiparallel and simultaneously the xanthene core of R6G is parallel with respect to SNP and exposes its flat part toward SNP. It suggested that the dye size has a negligible effect on the size of the measured dye–SNP complex, with the R6G having only <20% contribution to the measured size in 40 Å SNPs. This agrees with experimental results,^59^ where the measured size of the complex was in the range of uncertainty with the known size of the nanoparticle. The possibility of formation of an R6G layer on SNP is excluded due to dipole and geometric constraints; only one R6G molecule might adsorb on the SNP at a time. It is important to note that R6G contribution to the measured size increases with decreasing SNP size, as with decreasing size, the SNPs become less spherical and more curved and the measured size strongly depends on the measuring direction. As a result, the size contribution can be up to 30% in 20SNPs.

Finally, we studied the process of R6G dimerization using visual analysis and measured binding energies of R6G–40qSNP7 and R6G–40cSNP7 complexes and between two monomers, creating a dimer using SMD with constant-velocity pulling. We found that dimers can form both in solute and on the SNP surface, with dimerization in solute being favored over the latter one. For the 40SNP systems, there were 60 dimers formed in the solute and five dimers formed on the SNP matrix, all in pH 7. In 20SNP systems, 64 dimers were formed in the solute and 7 were formed on the SNP matrix. Furthermore, we have noticed that for 40SNPs, independently of the crystal structure, the average amount of dimers in the system drops at pH 12 when compared with pH 7. Both adsorption and dimerization impose geometric constraints on the R6G molecules due to the fact that it is nonsymmetric in the xanthene core plane, i.e., the two R6G in a dimer orient their xanthene cores in an antiparallel way, with the core planes facing each other. Same applies to R6G that is adsorbed to the SNP, where for the stable adsorption to occur, the xanthene core has to be parallel to the surface of the SNP. The geometrical constrains well explain the observed competition between R6G adsorption and dimerization, the fact that only monomeric R6G might form stable conglomerates with SNPs and R6G trimers are not stable, while the creation of higher oligomers is not possible. When looking at the 20qSNP case, we found that the average number of dimers increases from 4.75 at pH 7 to 5.75 at pH 12, and the time of existence also grows, from 20.97 to 21.17 ns, which suggests that in addition to the pH, the SNP size also has an impact on the process of dimerization. The binding energies obtained using SMD simulations confirmed our findings from the individual 40SNP studies, where qSNP had the strongest adsorption affinity and highest binding energy (∼1.08 eV), cSNPs had lower binding energy (∼0.36 eV), and the weakest were the interactions between two dimer components (∼0.27 eV).

The simulations performed in this work help to understand the mechanism of adsorption of cationic R6G to anionic SNPs. Due to the small size of the fluorescent dyes, it is impossible to experimentally determine the orientation of the dye on the surface of the SNPs; however, MD simulations have been successfully employed to investigate this in detail. It is important to mention that in the Classical Molecular Dynamics simulations used in the presented work, the partial charges of the atoms are defined when building the system and stay constant during the whole simulation. The use of Quantum MD to account for charge transfers and their fluctuations could be used for an additional layer of validation for the processes described herein. Nevertheless, based on our results as well as previous computational reports mentioned in this work, we are almost certain the charge fluctuations would not have a substantial impact on the R6G adsorption mechanism onto SNPs. The general interactions between R6G and SNPs studied in this work can be potentially extrapolated to other fluorescent dye interactions with both silica and other materials nanoparticles.

## Data Availability

Movies for most representative trajectories, SMD simulations, and R6G dimer desorption, dissociation, and temporal trimer; additional figures; R6G topology and parameters files, and tcl script used to extract COM distance between two entities. All data underpinning this publication, including inputs, parameter files, all other files required to reproduce the simulations and several trajectory files are openly available from the University of Strathclyde KnowledgeBase at 10.15129/68282cb9-2cc3-42bc-8bec-2090be5466b4. Any additional data needed will be shared on reasonable request to the corresponding author.

## Supporting Information Available

Theoretical consideration of methods and equations; R6G topology and parameters; custom TCL script that has been used to obtain the *XYZ* coordinates of any system component (PDF)
Most representative trajectory for the 40qSNP7 system (AVI)
Most representative trajectory for the 40qSNP12 system (AVI)
Most representative trajectory for the 40cSNP7 system (AVI)
Most representative trajectory for the 40cSNP12 system (AVI)
Most representative trajectory for the 20qSNP7 system (AVI)
Most representative trajectory for the 20qSNP12 system (AVI)
Most representative trajectory for the 20cSNP7 system (AVI)
Most representative trajectory for the 20cSNP12 system (AVI)
Dimer desorption from the SNP surface (AVI)
Dimer dissociation (breakage) on the SNP surface (AVI)
Temporary trimer formation and dissociation into a dimer and a monomer (AVI)
Constant-velocity SMD of the R6G dimer (AVI)
Constant-velocity SMD from the 40qSNP7 surface (AVI)
Constant-velocity SMD from the 40cSNP7 surface (AVI)

## Supplementary Material

ao3c06657_si_001.avi

ao3c06657_si_002.avi

ao3c06657_si_003.pdf

ao3c06657_si_004.avi

ao3c06657_si_005.avi

ao3c06657_si_006.avi

ao3c06657_si_007.avi

ao3c06657_si_008.avi

ao3c06657_si_009.avi

ao3c06657_si_010.avi

ao3c06657_si_011.avi

ao3c06657_si_012.avi

ao3c06657_si_013.avi

ao3c06657_si_014.avi

ao3c06657_si_015.avi
