# Supplementary material for: Impact of the Crystal Structure of Silica Nanoparticles on Rhodamine 6G Adsorption: A Molecular Dynamics Study
Source: ACS Omega. 2024 Jan 9;9(3):4123–36. doi: 10.1021/acsomega.3c06657 (PMC10809255; doi:10.1021/acsomega.3c06657)
Supplement: Supplementary file 3 — ao3c06657_si_003.pdf [file ao3c06657_si_003.pdf]

# Impact of Crystal Structure of Silica Nanoparticles on Rhodamine 6G Adsorption: A Molecular Dynamics Study

Daniel Doveiko[1], Karina Kubiak-Ossowska[2] and Yu Chen[1]

[1] Photophysics Group, Department of Physics, University of Strathclyde, Scottish Universities Physics Alliance, Glasgow G4 0NG, U.K.

[2] Department of Physics/Archie-West HPC, University of Strathclyde, 107 Rottenrow East, Glasgow G4 0NG, UK

# Supplementary Materials

Supplementary materials contain: (1) Theoretical consideration of methods and equations (2) Supplementary Figures, (3) a list of supplementary movies illustrating the main features described and discussed in the main text together with a brief description, (4) R6G Topology and Parameters, and (5) Custom TCL script that has been used to obtain the XYZ coordinates of any system component.

## 1. Theoretical consideration of methods and equations

### a) Electric Dipole Moment

Electric-dipole moments are well-defined for neutral molecules and are not very well described for usage in CHARGED systems, especially because the value depends on the selection. However, VMD uses a rather unusual way to deal with the selection issue by employing the following expression for a set of N atoms with partial charges $q_i$ and positions $r_i$:

$$p = \sum_{i=1}^{N} (q_i - q_0) r_i$$

where $q_0 = \frac{1}{N}\sum_{i=1}^{N} q_i$. This subtraction of $q_0$, which is the monopole component, makes the result independent of the choice of origin. Nonetheless, we checked the validity of this expression.

Let's consider

$$\underline{p} = \sum_{i=1}^{N}\left(q_i - {}^{Q}/_{N}\right)\underline{r_i} \qquad \left(Q = \sum_{i=1}^{N} q_i\right) \quad (1)$$

as our electric-dipole moment, defined here with $\underline{r} = 0$ as the origin of multipolar expansion.

Suppose now that we take $\underline{r} = \underline{a}$ to be the origin of the multipolar expansion instead. Our electric dipole moment is thus

$$\underline{p'} = \sum_{i=1}^{N}\left(q_i - {}^{Q}/_{N}\right)\left(\underline{r_i} - \underline{a}\right) \quad (2)$$

$$= \sum_{i=1}^{N}\left(q_i - {}^{Q}/_{N}\right)\underline{r_i} - \sum_{i=1}^{N}\left(q_i - {}^{Q}/_{N}\right)\underline{a} \quad (3)$$

$$= \underline{p} - \underline{a}\sum_{i=1}^{N}\left(q_i - {}^{Q}/_{N}\right) \quad (4)$$

$$= \underline{p} - \underline{a}\left(Q - {}^{NQ}/_{N}\right) \quad (5)$$

$$= \underline{p}, \quad (6)$$

matching our definition for $\underline{r} = 0$. Clearly, our electric-dipole moment is uniquely defined and independent of the origin of multipolar expansion.

## b) SMD binding energy

The formula is a simple potential energy of a spring formula but written in terms of the SMD measurable quantities:

$$dE = \left(F_0 + \frac{dF}{2}\right)\left(\frac{dF}{k}\right) \tag{7}$$

Let us assume that initially our virtual spring is at rest, hence $F_0 = 0$. We get:

$$dE = \frac{dF dF}{2\ k} \tag{8}$$

We also know, that:

$$dF = -kdx \tag{9}$$

This gives:

$$dE = \frac{(dF)^2}{2k} = \frac{k^2 dx^2}{2k} = \frac{1}{2} k dx^2 \tag{10}$$

which is simply the potential energy of the spring formula.

# 2. Supplementary Figures

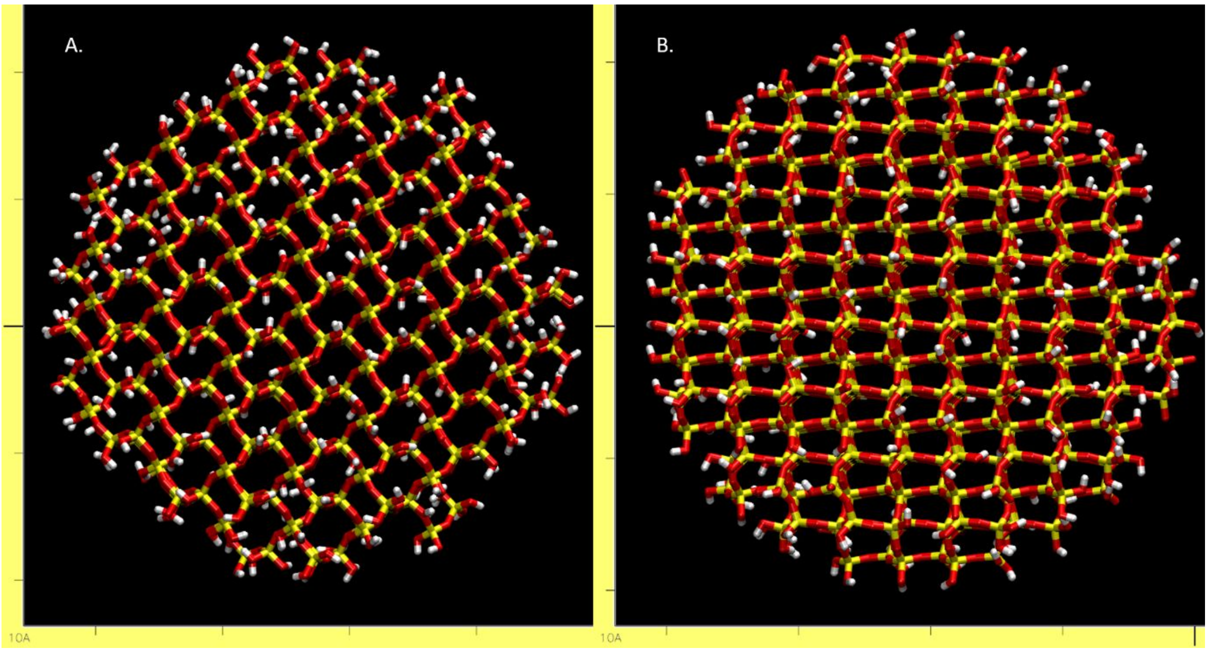


*Supplementary Figure S1. Crystal Structures of 40 Å SNPs. A. α-Quartz; B. α-Cristobalite.*

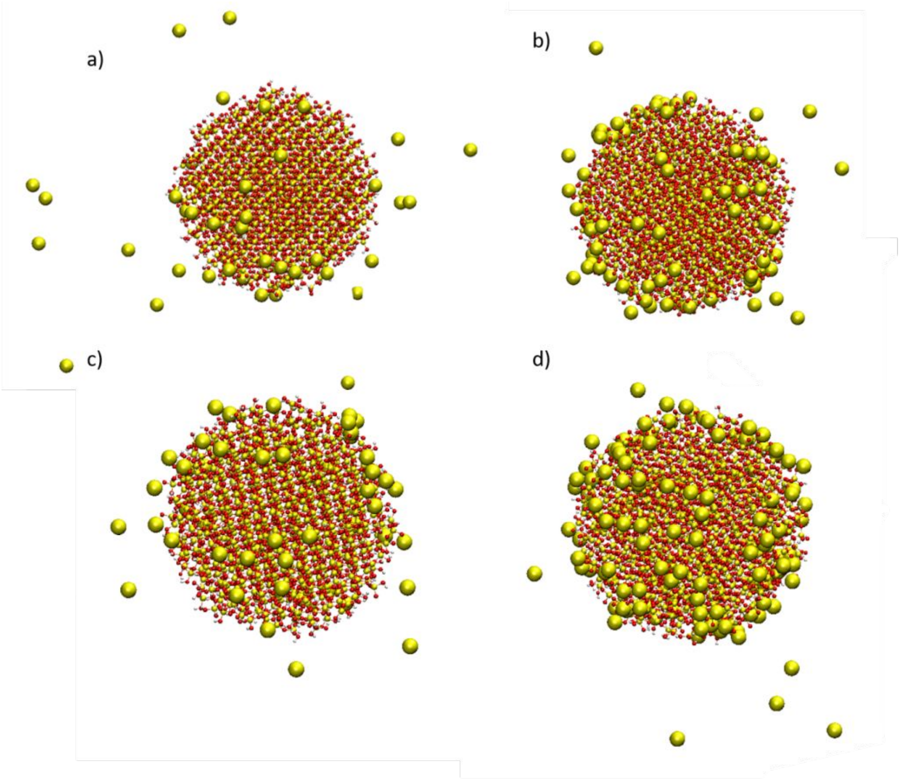


*Supplementary Figure S2. 40 Å SNP structures with ion layer: a) 40qSNP7; b) 40qSNP12; c) 40cSNP7; d) 40cSNP12.*

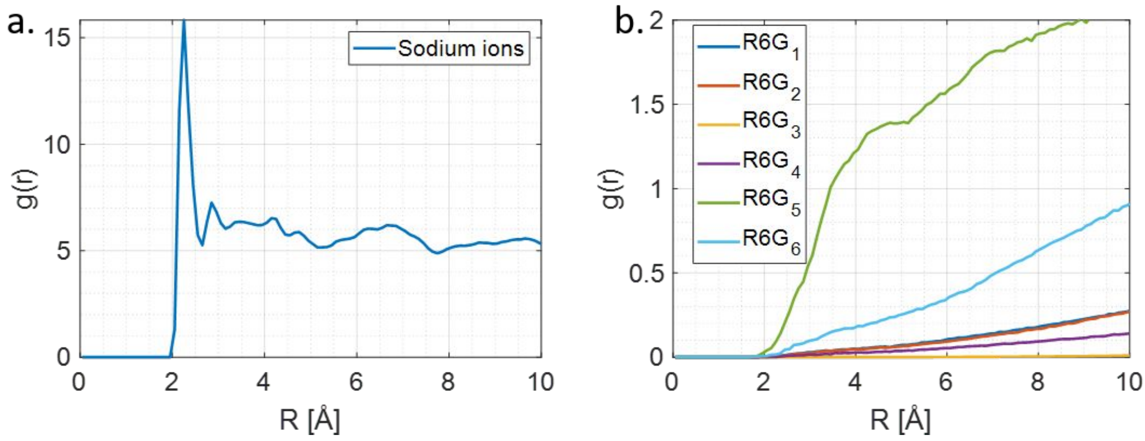


*Supplementary Figure S3. Radial Distribution Functions for 40qSNP7 system a) SNP-Na ions; b) SNP-all R6G molecules.*

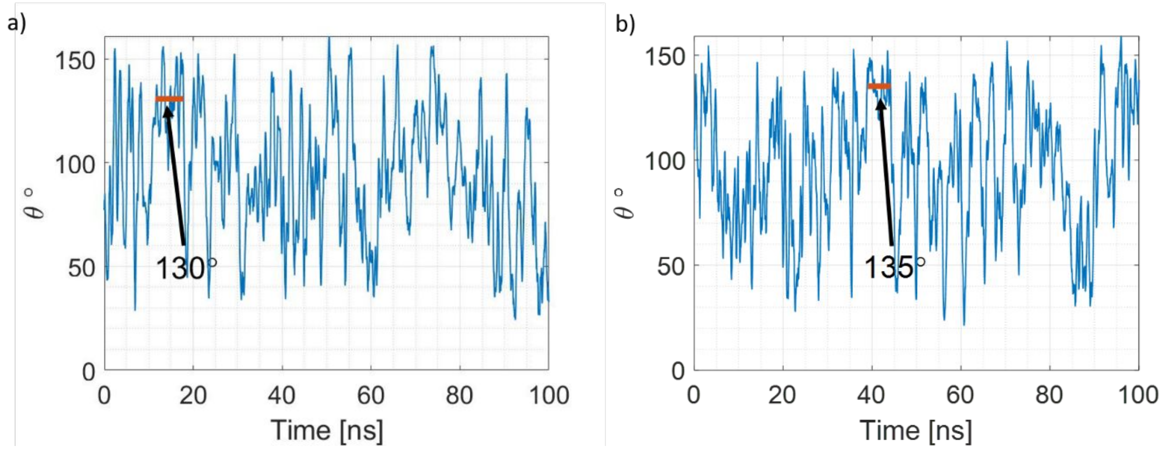


*Supplementary Figure S4. Angle between SNP and R6G dipole moments for 40cSNP7 system. a) Angle between dipole moments (ϑ) for R6G_3 for the R/A state in 40c SNP7; b) Angle between dipole moments (ϑ) for R6G_4 for the R/A state*

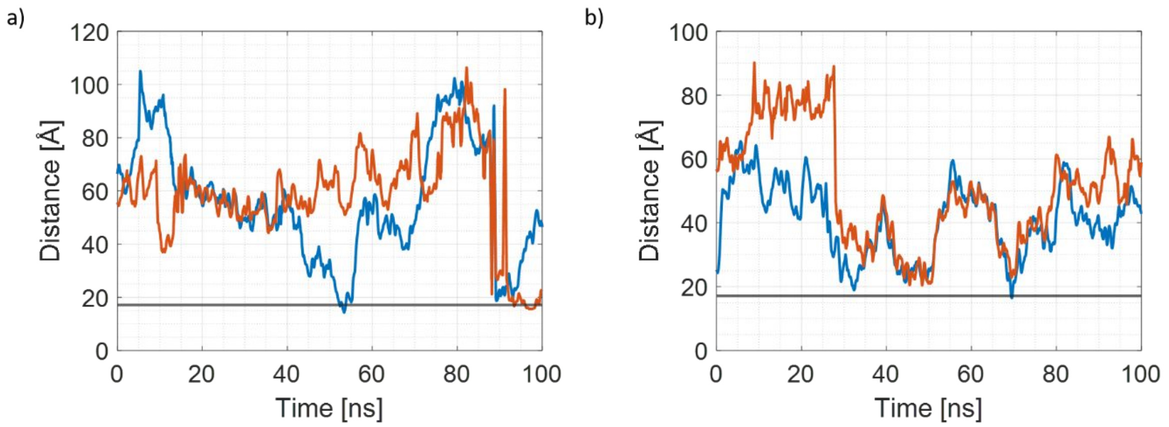


*Supplementary Figure S5. COM distance plots for 20cSNPs. a) Simplified COM distance plot for two best adsorbing R6G molecules, R6G_5 (blue) and R6G_6 (red) for 20cSNP7; b) Simplified COM distance plot for two best adsorbing R6G molecules, R6G_2 (blue) and R6G_5 (red) for 20cSNP12. The grey line marks the 5 Å distance from the SNP surface.*

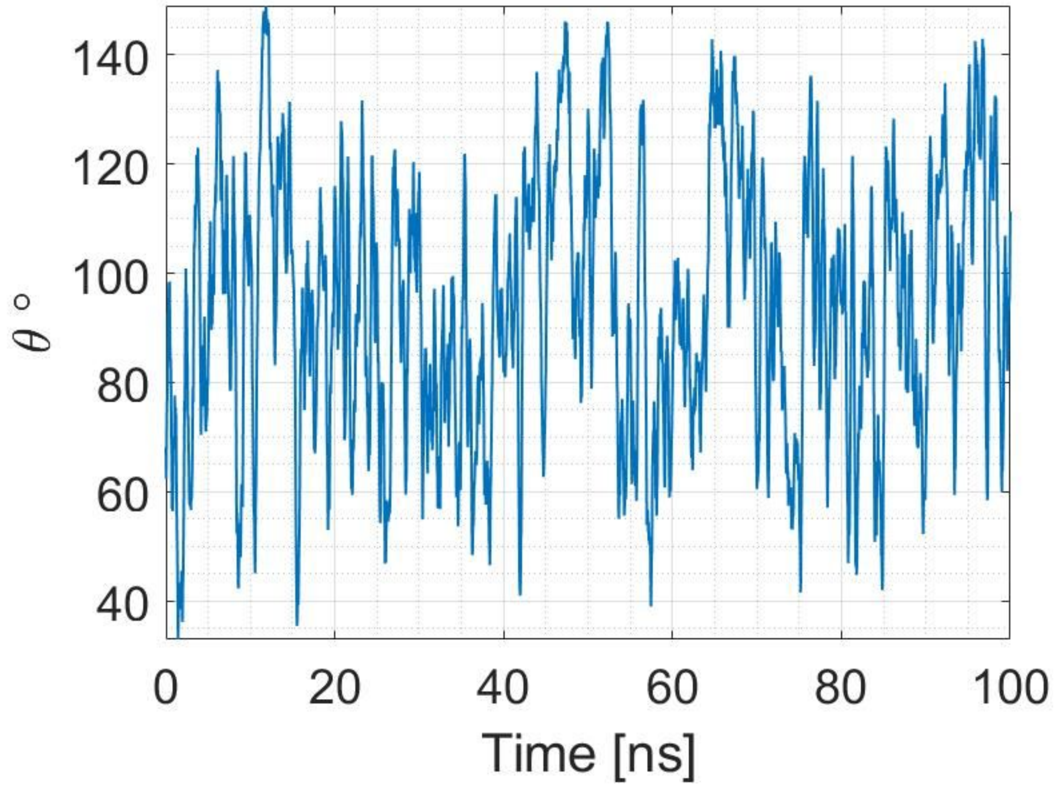


*Supplementary Figure S6. The angle between two R6G molecules in a dimer in the 20cSNP12 system, Details are discussed in section 3.7 of the main manuscript*

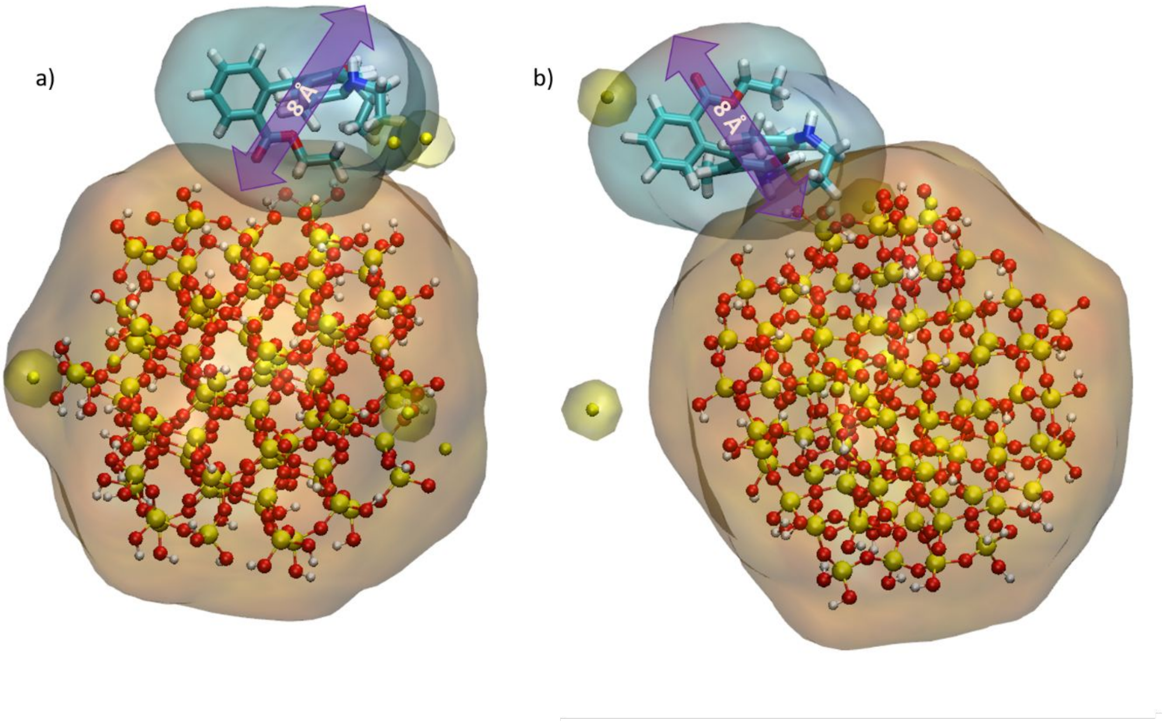


*Supplementary Figure S7. SNP-R6G structure. a) 20qSNP7-R6G complex; b) 20cSNP7-R6G complex*

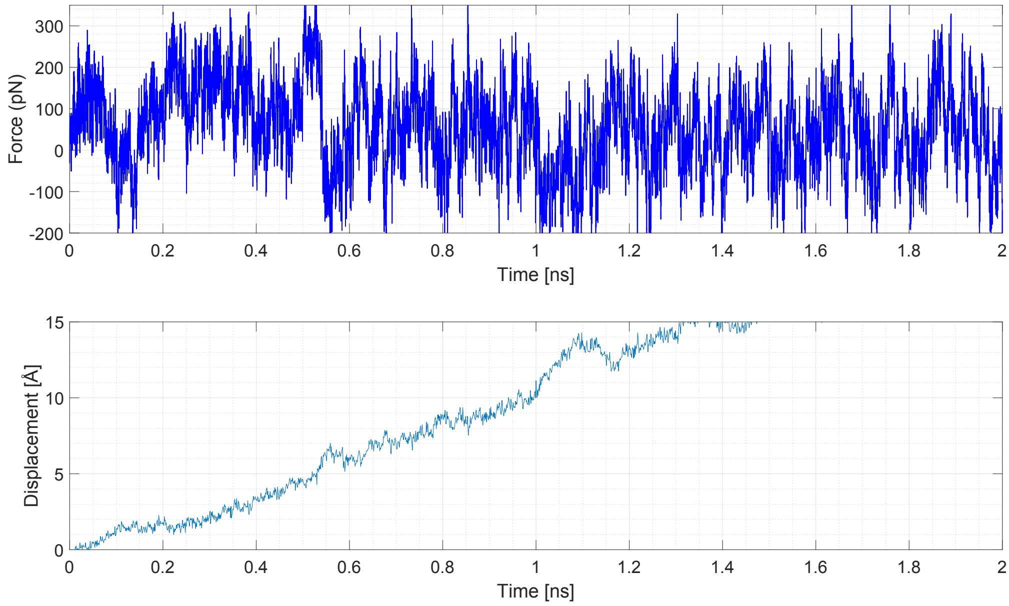


*Supplementary Figure S8. Force and Displacement as a function of time for the R6G pulled from the 40cSNP7 with constant velocity.*

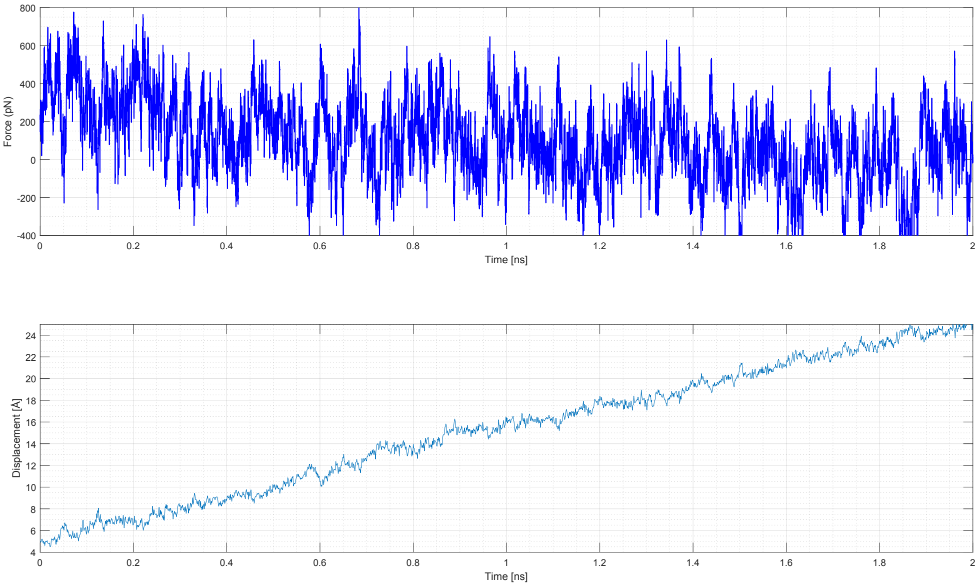


*Supplementary Figure S9. Force and Displacement as a function of time for the R6G dimer, where one of the components is pulled with constant velocity.*

# 3. Supplementary Movies

List of supplementary movies:

| ***Movie Name*** | ***Description*** | ***Representation*** |
|---|---|---|
| 40qSNP7.avi | The most representative trajectory for the 40qSNP7 system. | Only the two most representative R6G are shown using VdW representation (see Fig 5 for details). Water omitted for clarity |
| 40qSNP12.avi | The most representative trajectory for the 40qSNP12 system. | All R6G molecules are shown (See Figure 4 for details). |
| 40cSNP7.avi | The most representative trajectory for the 40cSNP7 system. | Only the two most representative R6G are shown using VdW representation (see Fig 8 for details). Water omitted for clarity |
| 40cSNP12.avi | The most representative trajectory for the 40cSNP12 system. | All R6G molecules are shown (See Figure 7 for details). |
| 20qSNP7.avi | The most representative trajectory for the 20qSNP7 system. | Only the two most representative R6G are shown using VdW representation (see Fig 9a for details). Water omitted for clarity |
| 20qSNP12.avi | The most representative trajectory for the 20qSNP12 system. | Only the two most representative R6G are shown using VdW representation (see Fig 9b for details). Water omitted for clarity |
| 20cSNP7.avi | The most representative trajectory for the 20cSNP7 system. | Only the two most representative R6G are shown using VdW representation (see Fig 10a for details). Water omitted for clarity |
| 20cSNP12.avi | The most representative trajectory for the 20cSNP12 system. | Only the two most representative R6G are shown using VdW representation (see Fig 10b for details). Water omitted for clarity |

| | | |
|---|---|---|
| DimerDesorption_40qSNP7.avi | Dimer Desorption from the SNP surface. | Only two R6G molecules forming a dimer are shown, which desorb from the SNP surface (as discussed in 3.7) |
| DimerDissociation_40qSNP7.avi | Dimer dissociation (breakage) on the SNP surface. | Three out of six R6G molecules are shown which participate in Dimer formation, which dissociates on the surface leaving behind an adsorbed R6G molecule (as discussed in 3.7) |
| R6GTrimer.avi | Temporary Trimer formation and dissociation into a dimer and a monomer | Three R6G molecules shown that temporarily form a trimer which dissociates into a monomer and a dimer (as discussed in 3.7) |
| DimerSMD.avi | Constant velocity SMD of R6G dimer | Two R6G molecules are shown, with one of them being fixed and the other being pulled with constant velocity |
| R6G_qSNP_SMD.avi | Constant velocity SMD from the 40qSNP7 surface | Only the qSNP and R6G of interest are shown in this movie. qSNP is fixed, while R6G is pulled from the surface with constant velocity |
| R6G_cSNP_SMD.avi | Constant velocity SMD from the 40cSNP7 surface | Only the cSNP and R6G of interest are shown in this movie. cSNP is fixed, while R6G is pulled from the surface with constant velocity |

# 4. R6G parameters

Below the contents of the topology and parameters files used for R6G are listed.

***Topology for R6G***

RESI R6G 1.000

GROUP ! CHARGE

ATOM N1 NG311 -0.423

ATOM N2 NG311 -0.423

ATOM C1 CG2R61 -0.442

ATOM C2 CG2R61 0.428

ATOM C3 CG2R61 -0.104

ATOM C4 CG2R61 -0.270

ATOM C5 CG2R61 0.083

ATOM C6 CG2R61 0.261

ATOM C7 CG2R67 0.135

ATOM C8 CG2R61 -0.104

ATOM C9 CG2R61 0.428

ATOM O1 OG3R60 -0.287

ATOM C10 CG2DC1 -0.442

ATOM C11 CG311 0.261

ATOM C12 CG2DC1 0.083

ATOM C13 CG2DC1 -0.270

ATOM C14 CG2R67 0.123

ATOM C15 CG2R61 -0.194

ATOM C16 CG2R61 -0.114

ATOM C17 CG2R61 -0.115

ATOM C18 CG2R61 -0.146

ATOM C19 CG2R61 -0.161

ATOM C20 CG331 -0.299

ATOM C21 CG331 -0.299

ATOM C22 CG2O2 0.822

ATOM O2 OG302 -0.489

ATOM O3 OG2D1 -0.568

ATOM C23 CG321 0.062

ATOM C24 CG331 -0.319

ATOM C25 CG321 0.062

ATOM C26 CG331 -0.319

ATOM C27 CG321 0.383

ATOM C28 CG331 -0.293

ATOM H1 HGPAM1 0.327

ATOM H2 HGPAM1 0.327

ATOM H3 HGR61 0.181

ATOM H4 HGR61 0.213

ATOM H5 HGA4 0.181

ATOM H7 HGA4 0.213

ATOM H8 HGR61 0.154

ATOM H9 HGR61 0.169

ATOM H10 HGR61 0.159

ATOM H11 HGR61 0.174

ATOM H12 HGA3 0.101

ATOM H13 HGA3 0.101

ATOM H14 HGA3 0.101

ATOM H15 HGA3 0.101

ATOM H16 HGA3 0.101

ATOM H17 HGA3 0.101

ATOM H18 HGA2 0.090

ATOM H19 HGA2 0.090

ATOM H20 HGA3 0.105

ATOM H21 HGA3 0.105

ATOM H22 HGA3 0.105

ATOM H23 HGA2 0.090

ATOM H24 HGA2 0.090

ATOM H25 HGA3 0.105

ATOM H26 HGA3 0.105

ATOM H27 HGA3 0.105

ATOM H28 HGA2 -0.003

ATOM H29 HGA2 -0.003

ATOM H30 HGA3 0.087

ATOM H31 HGA3 0.087

ATOM H32 HGA3 0.087

BOND H27 C26

BOND C26 H25

BOND C26 H26

BOND C26 C25

BOND H24 C25

BOND H23 C25

BOND C25 N1

BOND H3 C1

BOND N1 H1

BOND N1 C6

BOND C1 C6

BOND C1 C2

BOND H5 C10

BOND C6 C5

BOND H20 C24

BOND O1 C2

BOND O1 C9

BOND C2 C3

BOND C10 C9

BOND C10 C11

BOND H19 C23

BOND H2 N2

BOND C9 C8

BOND H17 C21

BOND C24 H22

BOND C24 C23

BOND C24 H21

BOND C5 C21

BOND C5 C4

BOND N2 C23

BOND N2 C11

BOND H15 C21

BOND C23 H18

BOND C21 H16

BOND C11 C12

BOND C3   C4

BOND C3   C7

BOND C4   H4

BOND C8   C7

BOND C8   C13

BOND C7   C14

BOND C12  C13

BOND C12  C20

BOND O3   C22

BOND C13  H7

BOND H29  C27

BOND H13  C20

BOND H12  C20

BOND C20  H14

BOND C14  C15

BOND C14  C19

BOND C22  C19

BOND C22  O2

BOND H8   C15

BOND H28  C27

BOND C27  O2

BOND C27  C28

BOND C15  C16

BOND C19  C18

BOND H32  C28

BOND C28  H30

BOND C28  H31

BOND C16  H9

BOND C16  C17

BOND C18  H11

BOND C18  C17

BOND C17  H10

IMPR C22 C19 O3 O2

END

***Parameters for R6G***

BONDS

CG2DC1 CG311 365.00 1.5020,

CG311 NG311 263.00 1.4740

ANGLES

CG2DC1 CG2DC1 CG311 48.00 123.50,

CG2R61 CG2DC1 CG311 48.44 127.34

CG311 CG2DC1 CG331 48.00 123.50

CG311 CG2DC1 HGA4 40.00 116.00

CG2DC1 CG2R61 CG2R67 36.00 120.00

CG2DC1 CG2R61 OG3R60 20.00 113.90

CG2O2 CG2R61 CG2R67 45.00 120.00

CG2DC1 CG311 CG2DC1 30.00 114.00

CG2DC1 CG311 NG311 78.00 110.00

CG2DC1 CG311 HGA1 45.00 111.50

NG311 CG311 HGA1 32.40 109.50 50.00 2.13000

CG311 NG311 CG321 40.50 109.60

CG311 NG311 HGPAM1 35.00 111.00

DIHEDRALS

CG2R61 CG2DC1 CG2DC1 CG311 0.5605 1 180.00

CG2R61 CG2DC1 CG2DC1 CG311 6.9692 2 180.00

CG311 CG2DC1 CG2DC1 HGA4 5.2000 2 180.00

CG2DC1 CG2DC1 CG2R61 CG2R67 0.7500 2 180.00

CG2DC1 CG2DC1 CG2R61 CG2R67 0.1900 4 0.00

CG311 CG2DC1 CG2R61 CG2R61 0.7500 2 180.00

CG311 CG2DC1 CG2R61 CG2R61 0.1900 4 0.00

CG311 CG2DC1 CG2R61 OG3R60 0.9539 2 180.00

CG311  CG2DC1 CG2R61 OG3R60     0.6267  4     0.00

HGA4   CG2DC1 CG2R61 CG2R67     0.6000  2   180.00

HGA4   CG2DC1 CG2R61 OG3R60     0.6000  2   180.00

CG2DC1 CG2DC1 CG311  CG2DC1     0.0000  3   180.00

CG2DC1 CG2DC1 CG311  NG311      0.7000  3     0.00

CG2DC1 CG2DC1 CG311  HGA1       0.0300  3     0.00

CG2R61 CG2DC1 CG311  CG2DC1     0.0000  3     0.00

CG2R61 CG2DC1 CG311  NG311      0.1900  3     0.00

CG2R61 CG2DC1 CG311  HGA1       0.6022  3   180.00

CG331  CG2DC1 CG311  CG2DC1     0.1900  3     0.00

CG331  CG2DC1 CG311  NG311      0.1900  3     0.00

CG331  CG2DC1 CG311  HGA1       0.1900  3     0.00

HGA4   CG2DC1 CG311  CG2DC1     0.2000  3     0.00

HGA4   CG2DC1 CG311  NG311      0.2000  3     0.00

HGA4   CG2DC1 CG311  HGA1       0.2000  3     0.00

CG311  CG2DC1 CG331  HGA3       0.1600  3     0.00

OG2D1  CG2O2  CG2R61 CG2R67     1.0250  2   180.00

OG302  CG2O2  CG2R61 CG2R67     0.8500  2   180.00

CG2R61 CG2O2  OG302  CG321      1.2500  1   180.00

CG2R61 CG2O2  OG302  CG321      1.5000  2   180.00

CG2R61 CG2O2  OG302  CG321      0.0500  6   180.00

CG2DC1 CG2R61 CG2R61 CG2DC1     3.1000  2   180.00

CG2DC1 CG2R61 CG2R61 CG2R67     3.1000  2   180.00

CG2DC1 CG2R61 CG2R61 OG3R60     2.4642  2   180.00

CG2R67 CG2R61 CG2R61 OG3R60     3.1000  2   180.00

CG331  CG2R61 CG2R61 NG311      2.4000  2   180.00

CG2DC1 CG2R61 CG2R67 CG2R61     3.1000  2   180.00

CG2DC1 CG2R61 CG2R67 CG2R67     3.1000  2   180.00

CG2O2  CG2R61 CG2R67 CG2R61     3.1000  2   180.00

CG2O2  CG2R61 CG2R67 CG2R67     3.1000  2   180.00

CG2DC1 CG2R61 OG3R60 CG2R61     0.7600  2     0.00

CG2DC1 CG311  NG311  CG321      1.1000  1   180.00

```
CG2DC1 CG311  NG311  CG321     0.6000  2   180.00
CG2DC1 CG311  NG311  CG321     0.5000  3     0.00
CG2DC1 CG311  NG311  HGPAM1    0.4800  3     0.00
HGA1   CG311  NG311  CG321     0.0000  3     0.00
HGA1   CG311  NG311  HGPAM1    0.0500  3     0.00
CG331  CG321  NG311  CG2R61    0.8000  1   180.00
CG331  CG321  NG311  CG2R61    1.1000  3     0.00
CG331  CG321  NG311  CG311     1.4200  1   180.00
CG331  CG321  NG311  CG311     0.8200  2     0.00
CG331  CG321  NG311  CG311     1.0200  3     0.00
CG331  CG321  NG311  HGPAM1    0.3000  3     0.00
HGA2   CG321  NG311  CG311     0.0000  3     0.00

IMPROPERS

END
```

# 5. TCL script

***COMcoord.tcl***

***Script that can be used to extract the COM coordinates of any selection***

```
#Daniel Doveiko April 6th 2023
#daniel.doveiko.2018@uni.strath.ac.uk
#script to save coordinates of 2 COM of any selected residues
#it creates a file coordinates.dat with the data
#to use: >source COMcoord.tcl
#            >measure_coordinates "protein and resid X" "protein and resid Y"

 proc measure_coordinates { selstring1 selstring2 {molID {top}} {weight
 {}}} {
```

```
set sel1 [atomselect $molID $selstring1]
set sel2 [atomselect $molID $selstring2]
#setlect 2 residues
set numframes [molinfo top get numframes]
#get number of frames
set output [open "coordinates.dat" w]
#opens file for writing
puts $output "Frame\tx-coord\ty-coord\tz-coord\tx-coord\ty-coord\tz-coord"
#puts headers on all columns
for {set i 0} {$i < $numframes} {incr i} {
#loop to go over all frames
animate goto $i
if {![llength $weight]} {
set cent_sel1 [measure center $sel1]
set cent_sel2 [measure center $sel2]
#if geometric center is in line with COM put coordinates
} else {
set cent_sel1 [measure center $sel1 weight $weight]
set cent_sel2 [measure center $sel2 weight $weight]
#if not measure COM and put coordinates
}
puts $output "$i\t$cent_sel1 $i\t$cent_sel2"
#puts measured coordinates in the file coordinates.dat
}
$sel1 delete
$sel2 delete
unset numframes
unset output
#clears variables
}
```
